# An epidemiological synthesis of emerging and re-emerging zoonotic disease threats in Cameroon, 2000–2022: a systematic review

**DOI:** 10.1016/j.ijregi.2022.12.001

**Published:** 2022-12-17

**Authors:** Nancy B. Tahmo, Frankline Sevidzem Wirsiy, Dum-Buo Nnamdi, Marcel Tongo, James V. Lawler, M. Jana Broadhurst, Charles S. Wondji, David M. Brett-Major

**Affiliations:** a University of Nebraska Medical Center, Omaha, Nebraska, USA; b Centre for Research in Infectious Diseases, Yaoundé, Cameroon; c Liverpool School of Tropical Medicine, Liverpool, UK; d Center for Research on Emerging and Re-Emerging Diseases (CREMER), Yaounde, Cameroon; e Institute of Medical Research and Study of Medicinal Plants (IMPM), Yaoundé, Cameroon; f Africa Centres for Disease Control and Prevention (Africa CDC), Addis Ababa, Ethiopia; g Amref Health Africa, Nairobi, Kenya

**Keywords:** Zoonosis, Emerging/re-emerging, Cameroon, Preparedness, Surveillance

## Abstract

**Introduction:**

Population factors such as urbanization, socio-economic, and environmental factors are driving forces for emerging/re-emerging zoonotic diseases in Cameroon. To inform preparedness and prioritization efforts, this study mapped out epidemiological data (including prevalence) of zoonotic diseases occurring in Cameroon between 2000 and 2022 by demographic factors.

**Methods:**

Following the PRISMA guidelines, a protocol was registered in the PROSPERO database (CRD42022333059). Independent reviewers searched the PubMed, Embase, CINAHL, Cochrane, and Scopus databases on May 30, 2022 for relevant articles; duplicates were removed, and the titles, abstracts, and full texts were screened to identify eligible articles.

**Results:**

Out of 4142 articles identified, 64 eligible articles were retrieved in the database search and an additional 12 from the cited literature (*N* = 76). Thirty-five unique zoonoses (viral, bacterial, and parasitic) were indexed, including Cameroon priority zoonoses: anthrax, bovine tuberculosis, Ebola and Marburg virus disease, highly pathogenic avian influenza, and rabies. The number of studies varied by region, ranging from 12 in the Far North to 32 in the Centre Region. The most reported were as follows: brucellosis (random-effects pooled estimate proportion (effect size), ES 0.05%, 95% confidence interval (CI) 0.03–0.07; *n* = 6), dengue (ES 0.13%, 95% CI 0.06–0.22; *n* = 12), avian and swine influenza virus (ES 0.10%, 95% CI 0.04–0.20; *n* = 8), and toxoplasmosis (ES 0.49%, 95% CI 0.35–0.63; *n* = 11), although *I*^2^ values were greater than 75%, thus there was high inter-study heterogeneity (*P* < 0.01).

**Conclusions:**

This understanding of the distribution of emerging and re-emerging zoonotic threats in Cameroon is vital to effective preventive and resource prioritization measures.

## Highlights

• From 2000 to 2022, 35 zoonoses (viral, bacterial, and parasitic) were reported in Cameroon.
• Human and environmental factors influence the dynamics of emerging/re-emerging zoonoses.
• Toxoplasmosis, dengue, brucellosis, and avian influenza were the most reported zoonoses.
• Hospital-based cross-sectional studies accounted for the majority of studies on humans.
• Community-level zoonoses risks and incidence/prevalence are underestimated.

## 1 Introduction

Over time, interactions between humans, animals, and their environment have changed significantly, leading to the heightened threat of infectious diseases, with some emerging and others re-emerging [1]. A majority (61%) of emerging infectious diseases have an animal origin, with animals either acting as reservoirs, vectors, or hosts of these pathogens that are transmissible to humans [2]. Zoonotic diseases involve a wide range of pathogens (bacteria, viruses, parasites, fungi, protozoa, and non-viral agents) [3], and their emergence and re-emergence are driven by population factors such as urbanization, food systems, sociocultural behaviours, economic influences, and environmental factors like agriculture, deforestation, and climate change [4,5]. Zoonotic diseases are associated with considerable morbidity and mortality, especially in low-resource settings that experience significant economic and societal losses as animal health and agricultural productivity are threatened [3]. In addition, zoonoses represent a serious global health security threat, as seen with the COVID-19 pandemic [1]. In resource-poor settings like Cameroon, detecting and responding to emerging infectious diseases, including zoonotic disease threats, can be daunting. It is important to devise effective and targeted interventions that assist prevention strategies through informing/educating public health leaders, veterinarians, environmental officers, communities, and other One Health actors [6].

Approximately 70% of residents in Cameroon are involved in small-scale agriculture. Cameroon has a geostrategic position in the Central African sub-region (Economic Community of Central African States, ECCAS) and is bordered by six countries including Nigeria and the Republic of the Congo. Coupled with its diverse landscape ranging from forests to plains, Cameroon is an ideal location for exploring risk from a broad range of zoonotic threats [7]. Also, a portion of the Congo Basin—a historical hotspot for zoonotic emergence—is located in the southern region of Cameroon. This risk is enhanced due to the typical hunting and butchering activities, consumption of bush animals, livestock husbandry, and frequent contact with wild animals [8,9].

The Congo Basin region is home to one of the largest and most biologically diverse rainforests in the world. It has a huge forest population of non-human primates and other animal reservoirs of potential and actual human pathogens. The region is believed to be the origin of several important emerging human viruses, including HIV, multiple arthropod-borne viruses (including chikungunya virus, Zika virus, Usutu virus, and Crimean–Congo haemorrhagic fever virus), monkeypox virus, and Ebola virus; a recent rhabdovirus called Bas-Congo virus also originated from this area [10]. Repeated sporadic outbreaks of Ebola and evidence that different HIV lineages have been independently transmitted to humans from their primary animal hosts multiple times in the past, indicate that there remains a persistent threat of not only the emergence in humans of novel viruses, but also the continuing re-emergence of globally relevant infectious pathogens.

Specific details of the burden of zoonotic diseases in Cameroon are not well understood. Epidemiological data on various zoonotic diseases across different health districts of Cameroon are abundant in the literature [11,12], and there are some systematic reviews of specific zoonotic diseases like leptospirosis and monkeypox [13,14], as well as groups of zoonotic diseases like those caused by bacterial and viral infections, from Africa as a whole [15]. The national zoonoses programme, unifying the approaches to the risk management of these threats, was established in 2014. In 2016, the programme produced a prioritization list for zoonotic diseases, five of which were set as top priorities using a semi-quantitative review approach: anthrax, bovine tuberculosis, Ebola and Marburg virus disease, highly pathogenic avian influenza, and rabies [7]. There is no comprehensive report of all zoonoses groups in Cameroon.

A synopsis of epidemiological data on zoonotic diseases would be a valuable step in understanding, through an evidence-based systematic search and synthesis of published findings, the threat pathogen exposures of Cameroonian communities related to zoonotic diseases. This would provide a basis to inform zoonotic disease prioritization and the identification of capacity and community awareness to prevent and prepare for zoonotic outbreaks. This systematic review and meta-analysis was conducted to determine the prevalence and distribution of priority and other zoonoses reported in humans and animals (with evidence of human transmission) in Cameroon between January 2000 and May 2022.

## 2 Materials and methods

### 2.1 Protocol registration

The protocol for this systematic review was developed following the Preferred Reporting Items for Systematic Reviews and Meta-analyses (PRISMA) guidelines [16] (Supplementary Material File S1); this review has been registered in the International Prospective Register of Systematic Reviews of the National Institute for Health Research (NIHR, UK) (PROSPERO ID: CRD42022333059).

### 2.2 Evidence gathering

The peer-reviewed literature was searched across six databases: Embase, PubMed, CINAHL, Scopus, African Index Medicus, and Cochrane. The search was conducted on May 30, 2022, using the following keywords: (emerging OR new OR re-emerging OR neglect*) AND (“zoonotic disease*” OR “animal disease*” OR vector-borne OR “cross-species transmission” OR “interspecies transmission” OR rabies OR anthrax OR “avian influenza” OR Ebola OR Marburg OR “Bovine tuberculosis” OR “Lassa Fever” “Yellow Fever” OR “Crimean-Congo Hemorrhagic Fever” OR Plague OR “*Yersinia pestis*” OR Leptospirosis OR “Nipah virus”) AND (Cameroon) (Supplementary Material File S2). Additional evidence was obtained from the grey literature, including reports from the World Health Organization (WHO) Disease Outbreak News, health district and country reports (District Health Information Software 2 and National Zoonoses Program database), and expert network consultations.

### 2.3 Eligibility criteria

Studies were included if they (1) reported zoonoses data and were published between January 1, 2000, and May 30, 2022; (2) involved human subjects of any age and animals related to human infections; (3) the study population was from any of the 10 regions of Cameroon (Adamawa, Centre, East, Far North, Littoral, North, North West, South, South West, and West regions); (4) the study was published in English; and (5) the article included an abstract and full text; this latter requirement was waived for the grey literature, such as district and government reports. All types of study designs except case reports were considered.

Unpublished studies, conference abstracts, protocols, reviews, letters, interventions (trials), studies conducted in other countries involving travellers from Cameroon, and animal diseases that are not known to be transmissible to humans were excluded.

### 2.4 Outcome measures

Outcomes abstracted from the retrieved articles included the following: for each represented zoonotic disease, when available, the total cases per year by district; case incidence by year, by district; disease prevalence by year (cross-sectional seroprevalence studies), by district; mortality rate by year among incident cases, by district; demographics of those directly impacted (cases and communities/region with case load); source of transmission and reservoir connected to the crossover event or vector; and for human-to-human or human–vector–human outbreaks defined as five or more cases within 30 days, the days between the first case and last case, days between the first case and having attained half of the cases, and estimated incubation period.

Additionally, narratives around spillover events were captured, where applicable (how the spillover happened and how it was controlled). From this, themes were identified and tagged.

### 2.5 Data abstraction and risk of bias

Selected articles were imported into Rayyan software, a semi-automated web and mobile-based tool for systematic reviews [17]. The software identified duplicates, and one of the authors verified and removed the duplicates. Two authors (N.B.T. and F.S.W.) independently screened the titles and abstracts, based on the eligibility criteria stated above to validate their selection, and screened the full text for selected articles. A third author (D.N.) resolved all conflicts after the title/abstract screening and again after full-text screening. The team developed a standardized data abstraction form coherent with the study objectives and outcome measures. This data abstraction template was pilot-tested on a subset of articles and then tested for face and construct validity.

The risk of bias across studies was assessed using the tool developed by Hoy and colleagues [18], a method that relies on the GRADE working group (Grades of Recommendation, Assessment, Development and Evaluation) and Cochrane approaches. The tool assesses external validity (items 1–4), for example *“Was some form of random selection used to select the sample, OR, was a census undertaken?”* and internal validity (items 5–10), for example *“Was the study instrument that measured the parameter of interest shown to have reliability and validity (if necessary)?”* for each study, where a score of 1 was given if the item was reported, 0 if it was not reported, and 0.5 for ‘no information’ ([18] p.4). N.B.T. and D.N. independently scored the studies, and the risk of bias was classified as low (8.5–10), moderate (5–8), or high (0–5.5).

### 2.6 Data synthesis

The management of abstracted data was accomplished in Microsoft Excel 2021. Meta-analysis was considered only when the level of bias and data harmonization were appropriate. An a priori power analysis for the test of significance of the variance component for each abstracted outcome was conducted for zoonotic diseases with at least five (*k*) reported studies, with the following parameters: degrees of freedom, df, *k* – 1 = 4; significance level, α = 0.5; the standard difference for the difference between disease proportions and mean, standard deviation (SD) = 6; and variance component, τ^2^ = 0.24 [19]. Thus, a power of 53.0% was obtained for a test of variation using five studies. Ngaya and colleagues [20] developed a Stata command (metaprop) for prevalence studies, which was employed in the present study to compute the 95% confidence intervals (CI) using the Wilson score interval, because some case counts were expected to be close to zero. Also, Freeman–Tukey double arcsine transformation was used to account for variance between studies, and the *I*^2^ statistic was used to assess the heterogeneity of the studies by zoonotic disease [20]. The power analysis was done using IBM SPSS Statistics version 26.0 (IBM Corp., Armonk, NY, USA) and the meta-analysis was conducted using Stata/BE 17.0 (StataCorp LLC, College Station, TX, USA).

ArcGIS Pro version 3.0.0 (Esri Inc.) was used to produce map visualizations of the data, and shapefiles were obtained through DIVA-GIS spatial data repository, with GADM version 1.0 (Database of Global Administrative Areas) as the primary source.

## 3 Results

**Figure 1:**
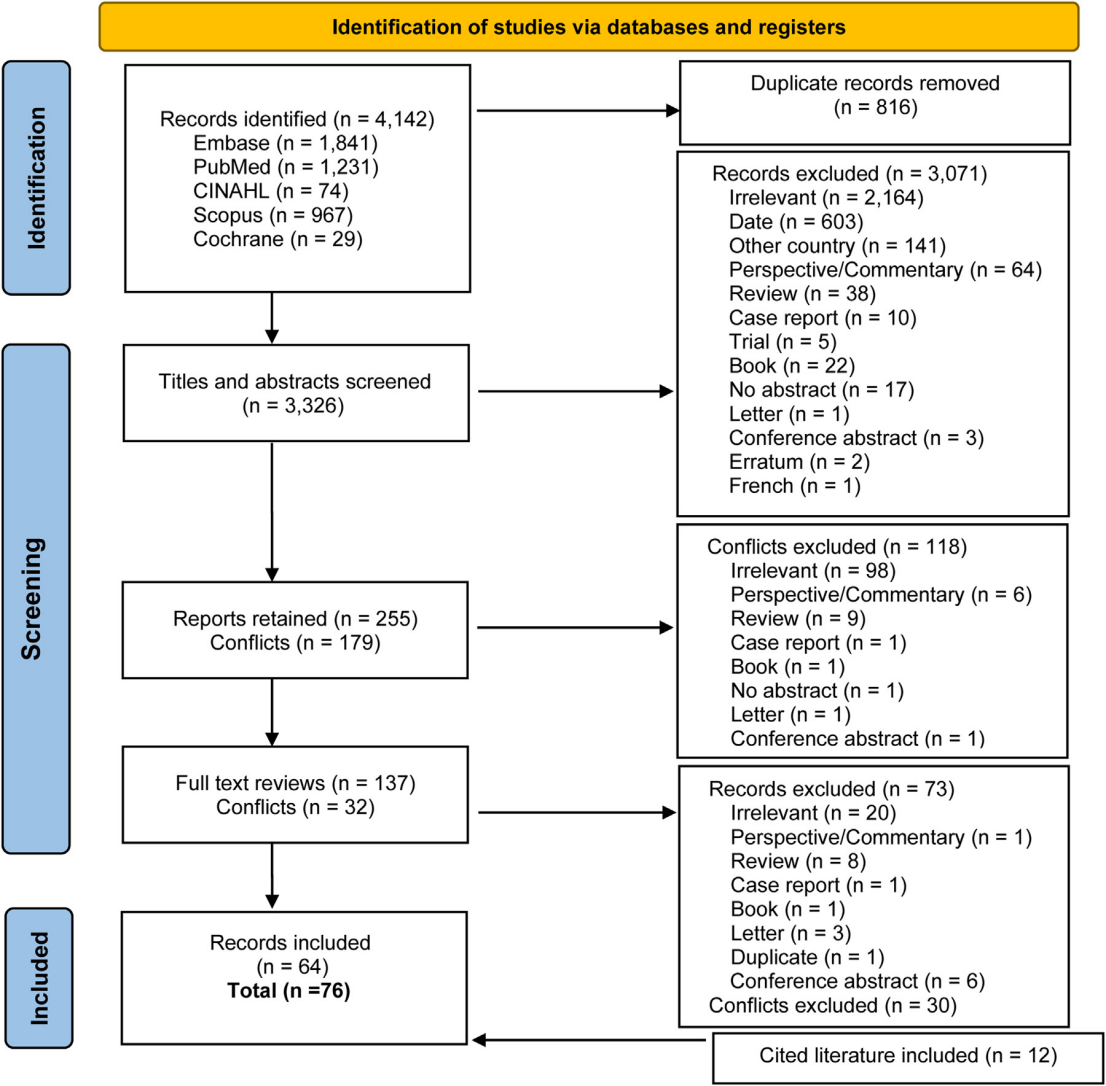
PRISMA flow diagram.

In total, 4142 articles were identified from the different databases (Embase = 1841; PubMed = 1231; CINAHL = 74; Scopus = 967; African Index Medicus = 0; and Cochrane = 29), of which 816 were duplicates. After screening, 64 articles were included for data abstraction and 12 were included from the literature cited in the articles retained for full-text screening, such as systematic reviews; overall, 76 studies were included for data synthesis (Figure 1).

### 3.1 Characteristics of the included studies

All of the studies employed a cross-sectional study design and involved both males and females. Fifty-two percent involved humans alone (0–80 years), 42% involved animals alone (cattle, non-human primates, sheep, goats, dogs, cavies, bats, birds, and swine), and 3% had both animal and humans as the study population. The studies spanned all 10 regions, however the number of studies varied by region: Adamawa = 17, Centre = 32, East = 18, Far North = 12, Littoral = 17, North = 13, North West = 21, South = 21, South West = 18, and West = 20. A majority of the studies targeted specific health districts and communities and were not necessarily representative of the regions. In total, 35 zoonotic diseases were reported: viral (Marburg, Ebola, avian and swine influenza, monkeypox, rabies, Nipah virus, enteroviruses, human T-lymphotropic virus, and other simian retrovirus diseases); bacterial (leptospirosis, Q fever, bovine tuberculosis, brucellosis, ehrlichiosis, Lyme disease, rickettsiosis); parasitic (myiasis, paragonimiasis, toxocariasis, toxoplasmosis); and other vector-borne viral zoonoses (Rift Valley fever, West Nile, dengue, Zika, yellow fever, Crimean–Congo haemorrhagic fever, chikungunya, Usutu, o'nyong-nyong fever, Wesselsbron, Semliki Forest, Tahyna, Sindbis, Spondweni, Middleburg virus infections) (Table 1, includes references).

**Table 1:** Characteristics of the included studies.

Citation (author, year)	Setting	Study period	Study population	Study design	Sampling	Infectious agent or disease
Viral zoonoses						
Njouom et al., 2008 [1]	North (Malape Garoua; peri-urban), Far North (Maroua and Vele; urban)	February–March 2006	246 ducks (domestic)	CS; three outbreaks	Simple random sampling; throat and cloacal swabs	Avian influenza A (H5N1)
Monamele et al., 2019 [2]	Adamawa, North West, South West, Centre, Littoral, East, South; market/farm, rural/ urban/peri-urban	May 2016–March 2017	147 poultry (domestic) and 663 exposed humans (presence in a farm/market; 0.5–80 years, mean 31 years, 25% female)	CS, outbreak	Simple random sampling; throat and cloacal swabs for ducks and swabs for humans	Avian influenza A (H5N1)
Wade et al., 2018a [3]	Adamawa, Centre, South, West; farm, peri-urban	May 2016–June 2017	4478 poultry (domestic)	CS, outbreak	Simple random sampling; swabs, eggs, tissues, dropping samples	Avian influenza A (H5N1) clade 2.3.2.1c virus
Wade et al., 2018b [4]	Far North; market, rural	January 2017	122 birds (peri-domestic)	CS, outbreak	Non-specified sampling; tracheal and cloacal swabs	Avian influenza A (H5N8)
Snoeck et al., 2015 [5]	North West; small-scale (backyard) farms, peri-urban	May–June 2011	197 pigs (2–26 months, mean 8.8 months)	CS	Simple random sampling; venous blood samples	Influenza A virus (H1N1)
Larison et al., 2014 [6]	Centre, West, North, and Far North; rural	December 2009–August 2012	325 pigs, 582 domestic birds, 1479 wild birds	CS	Stratified random sampling; nasal swabs and venous blood samples	Influenza A virus (pH1N1)
Njabo et al., 2012 [7]	Centre and North; villages and farms, rural	December 2009–April 2010	109 pigs	CS	Simple random sampling; nasal swabs and venous blood samples	Influenza A virus (pH1N1)
Njouom et al., 2012 [8]	Centre (5 sites), Littoral (3 sites), North (2 sites), West (4 sites); sentinel sites (private and public health clinics, urban)	January–December 2009	561 patients with influenza-like symptoms (1.2 months–75 years, median 6 years, 48.7% female)	CS	Convenience sampling; throat/ nasopharyngeal swab samples; inclusion criteria: sudden onset of fever (temperature >38°C) and cough or sore throat, with the onset of symptoms within the prior 5 days	Influenza virus, human rhinovirus, parainfluenza virus, enterovirus, human coronavirus (HCoV), human metapneumovirus (hMPV)
De Nys et al., 2018 [9]	National (10 regions)	November 2015–August 2017	2018 frugivorous and insectivorous wild bats	CS	Stratified random sampling; venous blood samples	Ebola virus (Zaire)
Steffen et al., 2019 [10]	South (Djoum, Ebolowa, Sangmelima); peri-urban	2011–2012	240 individuals	CS	Convenience sample; venous blood samples	Ebola virus (Zaire)
Harvala et al., 2011 [11]	South and East; rural/ peri-urban	2006–2009	54 wild-living NHPs (27 chimpanzees, 27 gorillas)	CS	Convenience sampling; faecal samples	Enteroviruses (types A–D)
Harvala et al., 2014 [12]	South and East; rural/ peri-urban	2010–2011	99 wild-living NHPs (chimpanzees, gorillas, bonobos)	CS	Convenience sampling; faecal samples	Enteroviruses (types A–D)
Sadeuh-Mba et al., 2014a [13]	Centre (Mfou HD: primate sanctuary and zoo); rural/urban	June 2006–October 2008	615 wild-born NHPs (99 zoo and 516 wild; chimpanzees, gorillas, monkeys)	CS	Convenience sampling; faecal samples	Enteroviruses (types A–D)
Van Nguyen et al., 2014 [14]	South and East; rural	2008–2012	113 wild-living NHPs (102 mandrills, 7 mangabeys, 4 monkeys)	CS	Convenience sampling; faecal samples	Enteroviruses (types A–H and J)
Zheng et al., 2010 [15]	East and West; rural	Not specified	402 primate hunters (18–64 years old, mean 36 years, 50.2% female, 5.1% Baka)	CS	Convenience sampling; venous blood samples	HTLV
Calattini et al., 2011 [16]	South (Campo); rural	2004–2008	35 family members of an HTLV-3-infected person (11–65 years, mean 39 years, 54.1% female); Bakola Pygmy tribe	CS	Family-tracing; venous blood samples	HTLV and Simian foamy virus
Guagliardo et al., 2020 [17]	Centre (Mfou HD: primate sanctuary, Metet, Nzdefidi, Ndangueng I, and Nkilzok I); rural	October 2017	125 individuals (45 primate sanctuary employees and 80 villagers) (18–83 years, median 37 years, 50.8% born after routine smallpox vaccination)	CS	Convenience sample; venous blood samples	Monkeypox virus (MPXV)
Steffen et al., 2020 [18]	South (Djoum, Ebolowa, Sangmelima); peri-urban	2011–2012	320 individuals	CS	Convenience sample; venous blood samples	Marburg virus
WHO, 2018 [19]	Centre (Biyem-Assi HD), South West (Akwaya HD), East (Bertoua HD), North West (Njikwa HD), Far North (Fotokol HD); urban	30 April–30 May 2018	1 month–58 years (median 13 years); 43.7% female	Outbreak	Non-specified sampling	Monkeypox virus (MPXV)
Pernet et al., 2014 [20]	East, West, South West, North West, Centre, Littoral; rural	February 2001–January 2003	44 bats and 487 individuals (227 bat-exposed (butchers, hunters) and 260 non-bat exposed)	CS	Simple random sampling; venous blood samples	Nipah virus
Courgnaud et al., 2004 [21]	Centre (Yaoundé) and South East; hub markets, villages, logging concessions; urban/rural	January 1999–July 2002	524 NHPs (328 bushmeat (monkeys, talapoins, gorillas) and 196 pet primates)	CS	Convenience sampling; venous blood samples	Primate T-cell lymphotropic virus (HTLV/STLV) type 1–3
Sadeuh-Mba et al., 2014b [22]	Centre, East, Littoral, North West, West; veterinary clinics, urban	2010–2013	91 dogs, 1 monkey, 1 pig	CS	Convenience sampling; brain specimens	Rabies virus (RABV)
Sadeuh-Mba et al., 2017 [23]	Centre, East, Littoral, North West, West, South West, South; urban/peri-urban	January 2010–December 2016	159 domestic dogs (65.4% from the Centre) + 1 cat, 1 cow, 1 monkey, 1 pig	CS	Convenience sampling; brain specimens	Rabies virus (RABV)
Betsem et al., 2011 [24]	South and East (Dja and Campo Maan nature reserves); rural/peri-urban	2006–2011	1321 individuals (356 Pygmies, mean age 43 years, 49.7% female; and 965 Bantus, mean age 51 years, 48.0% female) and 198 exposed individuals (bitten/scratched by NHP in their life) (78 Pygmies mean age 50 years, 97.4% male; and 120 Bantus, mean age 42 years, 95.8% male)	CS	Convenience sampling; venous blood samples	Simian foamy virus (SFV)
Calattini et al., 2004 [25]	South; rural	2000–2002	36 wild-caught apes and monkeys	CS	Convenience sampling; venous blood samples	Simian foamy virus (SFV)
Calattini et al., 2007 [26]	South; rural	2004–2005	102 individuals (2–80 years, mean age 40 years, 82.4% male); and 85 exposed individuals (bitten/scratched by NHPs); Bantu and Pygmy	CS	Convenience sampling; venous blood samples	Simian foamy virus (SFV)
Wolfe et al., 2004 [27]	South West, North West, West, Littoral, Centre, South, and East (9/17 villages from the Johns Hopkins HIV 2001–2003 survey); rural	2001–2003	1099/1800 individuals	CS	Stratified random sampling; venous blood samples	Simian foamy virus (SFV)
Bacterial zoonoses						
Awah Ndukum et al., 2010 [28]	North West (Bamenda), West (Dschang) abattoirs; urban	April–May 2008	35 292 cattle (33 835 Bamenda, 1460 Dschang)	CS	Simple random sampling; retropharyngeal/bronchial lymph nodes and liver tissues, venous blood samples	Bovine tuberculosis (*Mycobacterium bovis*)
Egbe et al., 2017 [29]	North West (Bamenda), Adamawa (Ngaoundere), North (Garoua), and Far North (Maroua); abattoirs/ market, urban	April 2012–October 2013	2346 cattle (1129 Bamenda, 935 Ngaoundere, 122 Garoua, 160 Maroua)	CS	Simple random sampling; venous blood and TB-like lesions/ retropharyngeal lymph node samples; sample size = Lorentz formula with 5% North West prevalence of lesions	Bovine tuberculosis (*Mycobacterium bovis*)
Awah-Ndukum et al., 2018a [30]	Adamawa (Tignere, Ngaoundere village, Meiganga, Tibati, and Banyo HDs) and North (Guider, Garoua, Poli, and Tchollire HDs); peri-urban	January–June 2014	1031 cattle (82 herds) (65% aged 5–8 years, 83% female)	CS	Stratified random sampling; venous blood samples; sample size = Lorentz formula using 16% prevalence of brucellosis; inclusion criteria: only herds with ≥10 head of cattle that are ≥2 years old and had spent ≥1 year in the area	Brucellosis
Awah-Ndukum et al., 2018b [31]	Adamawa (Ngaoundere); abattoir/ regional hospital; urban	August 2015–March 2016	590 cattle and 816 exposed humans (107 abattoir personnel and 709 pregnant women)	CS	Simple random sampling for animals and convenience for humans; venous blood samples; sample size = Lorentz formula with 5.4% prevalence	Brucellosis
Musallam et al., 2019 [32]	North West (Bamenda) and Adamawa (Ngaoundere); peri-urban	February 2017–January 2018	242 dairy cattle herds (100 Bamenda and 142 Ngaoundere)	CS	Simple random sampling; milk sample; sample size = Lorentz formula using 15% regional brucellosis prevalence	Brucellosis
Kamga et al., 2020 [33]	West (Noun), Centre (Yoko), South (Bipindi and Campo), South West (Fontem); peri-urban	December 2016–August 2018	855 cattle, 373 sheep, 452 goats, 33 dogs, 140 pigs	CS	Stratified random sampling; sample size = Lorentz formula with 5.2% bovine TB prevalence for cattle and 50% for the rest; venous blood samples	Brucellosis
Kelly et al., 2021 [34]	North West and Adamawa (Vina Division); peri-urban	January–November 2013	1558 cattle herds (750 NW and 748 VD pastoral cattle-71.9% female and 60 NW dairy cattle-98.3% female)	CS	Stratified random sampling; Sample size= Lorentz formula with 10% bovine TB prevalence; venous blood samples	Brucellosis, leptospirosis, Q fever
Scolamacchia et al., 2010 [35]	Adamawa; peri-urban	April–October 2000	1377 cattle (146 herds) (8 months–15 years, median 3 years, 70% female)	CS	Stratified, two-stage cluster sampling; jugular blood sample; sample size = from 50% foot and mouth disease seroprevalence	Brucellosis, leptospirosis, Q fever
Ndip et al., 2005 [36]	South West (Buea veterinary clinics); rural/ peri-urban/urban	March–October 2004	104 dogs (indoor confinement, outdoor, free dogs) (3 months–12 years, mean 3.4 years)	CS	Convenience sampling; venous blood samples	*Ehrlichia chaffeensis* and *Ehrlichia canis* (human monocytotropic ehrlichiosis (HME))
Ndip et al., 2009 [37]	South West (Buea and Tiko (Cameroon Development Corporation Central Tiko Clinic)); urban	January–June 2003	118 patients (65.3% female)	CS	Convenience sample; venous blood samples; inclusion criteria: febrile (fever 37.5–40.6°C at 1–9 days prior) and malaria and typhoid fever (*Salmonella*) negative	*Ehrlichia chaffeensis* (human monocytotropic ehrlichiosis (HME))
Abanda et al., 2019 [38]	North (Faro and Mayo-Rey), Adamawa (Vina and Faro et Deo), Far North (Mayo Tsanaga); urban	April 2014–June 2015	1260/1306 cattle (1–16 years, 76.9% female)	CS	Stratified random sampling; venous blood samples	Ehrlichiosis, rickettsiosis, Lyme disease
Ndip et al., 2004a [39]	South West (Cameroon Development Corporation Central Tiko Clinic and Mount Mary Health Centre, Buea); urban/peri-urban	January–June 2003	118 individuals (71% female)	CS	Convenience sampling; venous blood samples; inclusion criteria: malaria or typhoid fever negative	Spotted fever (rickettsiosis: *Rickettsia africae* and *R. conorii*)
Ndip et al., 2004b [40]	South West (Cameroon Development Corporation Central Tiko Clinic and Mount Mary Health Centre, Buea); urban/peri-urban	15 February–31 March 2001	234 febrile patients (88.5% ≥21 years, 60.7% female)	CS	Convenience sampling; venous blood samples; inclusion criteria: malaria or typhoid fever negative	Spotted fever (rickettsiosis: *Rickettsia africae* and *R. conorii*), chikungunya (CHIKV), yellow fever, dengue (DENV), West Nile virus (WNV), Spondweni virus
Parasitic zoonoses						
Kouam et al., 2015 [41]	West (Menoua); farms; rural	March 2013–February 2014	83 farms (62 privately owned farms and 21 research farms)	CS	Snow-ball sampling; faecal samples	Myiasis (*Cordylobia anthropophaga*)
Kouam et al., 2017 [42]	West and North West (Menoua and Bamboutos); farms, rural	June–July 2014	397 cavies/guinea pigs (123 farms)	CS	Snow-ball sampling; skin larvae samples from lesions; sample size = Lorentz formula with 50% prevalence	Myiasis (*Cordylobia anthropophaga*)
Nkouawa et al., 2009 [43]	South West (Tombel HD); rural	January 2004–February 2006	168 people who eat crabs (4–78 years, 15.2 ± 8.2 years for males and 12.9 ± 5.9 years for females)	CS	Convenience sampling; venous blood samples, sputum, and faecal samples; inclusion criteria: experienced symptoms such as cough, haemoptysis, headache, epilepsy, and chest pain, and whether they consumed raw and/or undercooked crabs	Paragonimiasis
Moyou-Somo et al., 2003 [44]	South West (Kumba primary school); peri-urban	March–July 2001	309/1482 pupils with signs of paragonimiasis (4–17 years, 9.79 ± 2.83 years in males and 9.04 ±2.47 years in females)	CS	Convenience sampling; stool/sputum samples	Paragonimiasis
Nkouawa et al., 2010 [45]	South West (Tombel HD); rural	January 2004–February 2006	168/188 people who eat crabs (4–78 years, 15.2 ± 8.2 years for males and 12.9 ± 5.9 for females)	CS	Convenience sampling; venous blood samples	Toxocariasis and paragonimiasis
Abongwa et al., 2019 [46]	North West (Bamenda Regional Hospital); urban	May–December 2017	606/683 pregnant women (14–45 years, 27.3 ± 5.3 years)	CS	Convenience sampling; venous blood samples; sample size: Lorentz formula using 54.5% regional HIV prevalence	Toxoplasmosis
Achonduh-Atijegbe et al., 2016 [47]	Centre (Nkolbisson HD outpatient facility); urban	February–April 2014	315 children (0.5–15 years, 5.8 ± 3.8 years, 50.2% female)	CS	Convenience sampling; venous blood samples; sample size = based on 38% malaria prevalence; inclusion criteria: within the age limit, a history of fever in the preceding 24 h or axillary temperature ≥38°C at the consultation	Toxoplasmosis
Assob et al., 2011 [48]	Centre (Yaoundé Teaching Hospital); urban	February–May 2010	133 HIV/AIDS patients (57.14% age 21–30 years)	CS	Convenience sampling; venous blood samples	Toxoplasmosis
Ayeah et al., 2022 [49]	Centre (Biyem-Assi District Hospital and CASS Nkoldongo); urban	June 2019–May 2020	259 neonates and 300/310 pregnant women (15–49 years, 28.05 ± 5.83 years, 2^nd^–3^rd^ trimester) (16.5% dropout due to complications like stillbirths)	CS	Convenience sampling; venous blood samples from mothers and cord blood from newborn umbilical cord; sample size = Cochran's formula from 65.5% prevalence	Toxoplasmosis
Kouitcheu et al., 2018 [50]	West (Penka-Michel, Menoua); rural	April–June 2014	643 pregnant women (15–50 years, 27.1 ± 2.51 years, 9.8% 1^st^ trimester, 39.5% 2^nd^, and 50.7% 3^rd^)	CS	Convenience sampling; venous blood samples	Toxoplasmosis
Ndamukong-Nyanga et al., 2021 [51]	Centre (Biyem-Assi District Hospital); urban	May–November 2019	232/300 pregnant women (52.16% age 21–30 years)	CS	Convenience sampling; venous blood samples; sample size = Lorentz formula using 23% toxoplasmosis prevalence	Toxoplasmosis
Nguefack et al., 2016 [52]	Littoral (Douala General Hospital + 2 clinics); urban	10 January–30 April 2015	327/402 pregnant women (31 ± 5 years)	CS	Convenience sampling; venous blood samples	Toxoplasmosis
Nguemaïm et al., 2020 [53]	North West (Bamenda Regional Hospital); urban	1 January–31 August	127 pregnant women (14–50 years, 27.4 ± 6.21 years, 50.4% in 1^st^ trimester and 44.2% in 2^nd^ trimester)	CS	Simple random sampling; venous blood samples	Toxoplasmosis
Njunda et al., 2011 [54]	Centre (Yaoundé Teaching Hospital); urban	May–June 2008	110 pregnant women (18–41 years, 5.8 ± 3.8 years, 36.4% in 1^st^ trimester and 60% in 2^nd^ trimester)	CS	Convenience sampling; venous blood samples	Toxoplasmosis
Guemgne Todjom et al., 2019 [55]	West (Mbouo-Banjoun Protestant Hospital); urban	June–September 2016	200 pregnant women (18% HIV-positive)	CS	Convenience sampling; venous blood samples	Toxoplasmosis
Wam et al., 2016 [56]	North West (St Martin de Porress Hospital Njinikom); rural	August–December 2014	178/350 women (15–49 years, 31.1 ± 8.1 years, nurses and patients)	CS	Convenience sample; venous blood samples	Toxoplasmosis
Other vector-borne viral zoonoses						
Fokam et al., 2010 [57]	South West (Fako Division Provincial Hospital Annex, Mount Mary Health Centre, Cameroon Development Corporation Central Clinic); urban	Not specified	102 febrile patients (42.2% female)	CS	Convenience sample; venous blood samples	Chikungunya (CHIKV), yellow fever virus (YFV), o'nyong'nyong (ONNV) viruses, dengue virus (DENV-1 to 4), Wesselsbron virus (WSLV), Zika virus, West Nile virus (WNV), Rift Valley fever virus (RVFV), Semliki Forest virus (SFV), Sindbis virus (SINDV); Middleburg virus (MIDV), Tahyna orthobunyavirus (TAHV)
Demanou et al., 2010 [58]	North West (Kumbo HD: Ngehndzen, Ndzeru, Tasaï); rural	November 18–26, 2006	105 patients (95% suspected cases) (50 ± 17.5 years, 44% female)	CS	Convenience sample; venous blood samples	Chikungunya virus (CHIKV), o'nyong'nyong virus (ONNV), dengue virus (DENV)
Kuniholm et al., 2006 [59]	South West, North West, West, Littoral, Central, South, and East (9/17 villages from the Johns Hopkins HIV 2001–2003 survey); rural	2001–2003	256 individuals (≥16 years, 48.4% age 16–35 years, 44.1% female)	CS	Stratified random sampling; venous blood samples	Chikungunya virus (CHIKV), West Nile virus (WNV), o'nyong'nyong virus (ONNV), yellow fever virus (YFV), dengue-2 (DENV-2), Sindbis virus (SINDV), Tahyna orthobunyavirus (TAHV)
González Gordon et al., 2022 [60]	North West and Adamawa (Vina Division); peri-urban	January–November 2013	1662 cattle (1498 pastoral cattle (71.2% female) and 164 dairy cattle)	CS	Stratified random sampling; venous blood samples; sample size = Lorentz formula with 10% bovine TB prevalence	Crimean–Congo haemorrhagic fever virus (CCHFV)
Demanou et al., 2014 [61]	Littoral (Douala), North (Garoua) and Centre (Yaoundé); urban	September 2006–December 2007	2030 individuals (728 North, 50.8% female; 675 Littoral, 55.3% female; 640 Centre, 60.2% female) (2–99 years, median 28 years)	CS	Random cluster sampling; venous blood samples	Dengue virus (DENV 1, 2, 3, and 4)
Raulino et al., 2022 [62]	Centre (Obala and Yaoundé), South (Campo), and East (Mambele); urban/rural	2016–2019	1376 bats (1266 frugivorous, 95 insectivorous, 15 indeterminate)	CS	Convenience sample; venous blood samples	Dengue virus (DENV 1–4), Zika virus (ZIKV), West Nile virus (WNV), Usutu virus, chikungunya virus (CHIKV), and o'nyong'nyong virus (ONNV)
Galani et al., 2021 [63]	Adamawa (Ngaoundere); regional hospital; urban	30 October 2019–15 January 2020	174 febrile patients (7 months–80 years, mean 23.17 years, 48.9% female)	CS	Convenience sampling; venous blood samples; sample size = Lorentz formula using 4.2% national malaria–dengue co-infection rate; inclusion criteria: febrile (fever, headache, chills, joint/abdominal pain)	Dengue virus (DENV)
Tchetgna et al., 2021 [64]	Littoral (4 Douala public hospitals); urban	July–December 2020	320 patients (29 ± 17 years, 80% female)	CS	Convenience sampling; venous blood samples; inclusion criteria: acute febrile syndrome (38°C <7 days duration) and >3 years old	Dengue virus (DENV)
Tchuandom et al., 2018 [65]	Far North (Kaelé), Adamawa (Bankim), Centre (Ntui, Yaoundé and Bafia), Littoral (Edéa and Douala), West (Bangangté, Foumban and Dschang); public hospitals; urban/peri-urban	March 2016–April 2017	961 febrile children ≤15 years (7.1 ± 2.9 years, 48.5% female)	CS	Systematic sampling technique; venous blood samples; sample size = Lorentz formula with 50% prevalence; inclusion criteria: temperature ≥38°C with at least one of specific symptoms (fever, headache, rash, vomiting, and joint pain)	Dengue virus (DENV)
Tchuandom et al., 2019 [66]	Centre, Littoral, West, Adamawa, Far North; public hospitals; urban/peri-urban	2018	961 febrile children ≤15 years (7.1 ± 2.9 years, 48.6% female)	CS	Systematic random sampling; venous blood samples; sample size = Lorentz formula; inclusion criteria: oral temperature ≥38°C, fever <7 days with at least one of specific symptoms	Dengue virus (DENV)
Tchuandom et al., 2020 [67]	Centre (Yaoundé-Jamot Hospital); urban	March–August 2019	310 blood donors (18–57 years, 29.4 ± 7.8 years, 8.4% female)	CS	Convenience sample; venous blood samples; sample size = Lorentz formula with 24.2% prevalence	Dengue virus (DENV)
Yousseu et al., 2018 [68]	Littoral (Douala New-Bell District Hospital); urban	March–April 2017	114 febrile patients (40.4% age 0–19 years, 33.3% age 20–39 years, 36.8% female)	CS	Convenience sampling; venous blood samples	Dengue virus (DENV), chikungunya virus (CHIKV), Zika viruses (ZIKV)
Mouiche et al., 2020 [69]	South (Meyomessala and Sangmelima District Hospitals); urban	September 2017–September 2018	629/649 individuals (434 patients, 50% female; and 215 community members, 31.2% female)	CS	Convenience sample; venous blood samples; inclusion criteria: fever ≥38°C and symptoms (haemorrhage, encephalitis, diarrhoea, vomiting)	Dengue virus (DENV-1)
Nemg Simo et al., 2018 [70]	South (Kribi HD); rural	June 21–25, 2017	91 febrile individuals (50.5% female)	CS, outbreak	Convenience sampling; venous blood samples	Dengue virus (DENV-1)
Ebogo-Belobo et al., 2022 [71]	Centre (Yaoundé); market slaughterhouse; urban	March 4–20, 2020	47 sheep and 144 goats (60.2% female)	CS	Non-specified sampling; venous blood samples	Rift Valley fever virus (RVFV)
Rissmann et al., 2017 [72]	Far North, North, Adamawa, North West, West, Centre, South West, Littoral and South; urban/peri-urban/rural	2013–2014	921 sheep and goats and 1032 cattle	CS	Stratified random sampling; venous blood samples; sample size = Lorentz formula using a prevalence of 1%	Rift Valley fever virus (RVFV)
Sadeuh-Mba et al., 2018 [73]	East (Abong Mbang, Lomié, Messok, Mindourou); rural	2005–2012	137 individuals (44.4 ± 16.4 years, 41.7% female, Pygmies)	CS	Simple random sampling; venous blood samples	Rift Valley fever virus (RVFV), Crimean–Congo haemorrhagic fever virus (CCHFV)
Poueme et al., 2019 [74]	North (Bénoué, Mayo-Rey, Faro); urban	January 2016–January 2017	355 goats (37 herds) and 325 sheep (28 herds, 1–3 years)	CS	Stratified random sampling; venous blood samples; sample size = Lorentz formula with prevalence = 45%	Rift Valley fever virus (RVFV)
Nemg Simo et al., 2022 [75]	National (all 197 HDs)	January 2010–December 2020	21261 suspected human cases (19.2 ± 16.9 years)	CS	Convenience sample; venous blood samples	Yellow fever
Gake et al., 2017 [76]	Far North (Maroua), North (Garoua), Adamawa (Ngaoundéré), East (Bertoua), Centre (Yaoundé), Littoral (Douala); urban	August–October 2015	1084 blood donors	CS	Convenience sample; venous blood samples	Zika virus (ZIKV)
Citation (Author, year)	Infectious agent or disease	Point/period prevalence by year/ location (%)	Mortality rate by year/ location (%)	Source of transmission and/or reservoir connected	Index case description	Diagnostic tool + case definition reference
Viral zoonoses						
Njouom et al., 2008 [1]	Avian influenza A (H5N1)	1.2% H5+ (66.7% N1+)	86.2%: 50 dead (February 2006, Maroua, Far North); 1 dead (March 2006, North); 43 dead (March 2006, Vele, Far North)	H5N1 isolate shown to be closely related to the H5N1 isolates from Northern Nigeria, Sudan, and Ivory Coast suggesting a common virus progenitor; introduction in Cameroon is unclear	58 index cases (Feb 21, 2006; Maroua, Far North); 1 index case (Mar 9, 2006; Lake Malape, Garoua); third outbreak (Mar 16, 2006, Far North)	RT-PCR
Monamele et al., 2019 [2]	Avian influenza A (H5N1)	Ducks total: 39.5% by RT-qPCR (58.6% A(H5N1)); Centre: 32.2% (78.9% A(H5N1)); South: 45.8% (54.4% A(H5N1)); West: 50% (100% A(H5N1)); Adamawa and East: 0%; North West: 33.3% (0% A(H5N1)) Humans total: 2.3% H5N1-positive and 1.7% H3N2-positive			Index case: May 24, 2016	RT-qPCR
Wade et al., 2018a [3]	Avian influenza A (H5N1) clade 2.3.2.1c virus	5.0% by avian flu rapid test; 7.3% positive for M and H5 genes; 0% positive eggs	138 252 dead birds (44 451 deaths due to infection and 93 801 culled)	The topology of the phylogeny based on the haemagglutinin gene segment indicated that the causative H5N1 viruses fell within the genetic clade 2.3.2.1c, within the same group as the A/H5N1 viruses collected in Niger in 2015 and 2016	In May 2016, HPAIV A/H5N1 was detected in Cameroon in an industrial poultry farm at Mvog-Betsi, Yaoundé (Centre region), with a recorded sudden increase in deaths among chickens, and an overall mortality rate of 75% In total, 21 outbreaks were confirmed from May 2016 to March 2017 (six in the Centre, six in the West, eight in the South, and one in the Adamawa regions)	FASTest AIV antigen test + real-time RT-PCR
Wade et al., 2018b [4]	Avian influenza A (H5N8)	4.1% positive (1 pigeon, 1 chicken, 2 guinea fowls, 1 duck)	103/107 of the peafowls died in 2 weeks, 24/24 fowls died	Sequences of the 2 viruses from Maroua market were identical for all the available gene segments and clustered with viruses collected in Asia, Europe, and Egypt	First death was reported on January 2 in peafowls	Real time RT-PCR
Snoeck et al., 2015 [5]	Influenza A virus (H1N1)	5.6% H1N1-positive (20.7% of herds)				Virus microneutralization (VN) assays on Madin–Darby canine kidney
Larison et al., 2014 [6]	Influenza A virus (pH1N1)	0.62% PCR-positive swine; 0% type A ELISA-positive birds				Real-time RT-PCR for swabs and competitive ELISA for sera + haemagglutination–inhibition assay
Njabo et al., 2012 [7]	Influenza A virus (pH1N1)	1.8% PCR-positive swabs and 28% seropositive; 92.6% cross-reactivity with A/H1N1/2009				Real-time RT-PCR for swabs and competitive ELISA serological assay
Njouom et al., 2012 [8]	Influenza virus, human rhinovirus, parainfluenza virus, enterovirus, human coronavirus (HCoV), human metapneumovirus (hMPV)	Influenza virus: 28.2% (97.5% influenza A: 92.2% A[H3N2], 5.2% A[H1N1]pdm09, and 2.6% A[N1H1]); human rhinovirus: 17.8%; parainfluenza virus types 1–4: 7.5%; enterovirus: 5.9%; human coronavirus OC43, 229E, NL63, and HKU1: 5.3%; human metapneumovirus: 5.0%				Real-time and multiplex RT-PCR
De Nys et al., 2018 [9]	Ebola virus (Zaire)	Insectivorous bat: 1–6% [0.4–2.3] and frugivorous bat: 1–9% [0.6–5.7]				Luminex assay + RT-PCR
Steffen et al., 2019 [10]	Ebola virus (Zaire)	1.3% [1.3–3.8] seropositive (1.7% EBOV neut-positive, 3.3% EBOV LIPS-positive (33.3% ELISA-positive))				Pseudotype microneutralization assay (EBOV neut) and luciferase immunoprecipitation system assay (EBOV LIPS) + confirmatory Zaire IgG ELISA
Harvala et al., 2011 [11]	Enteroviruses (types A–D)	14.8% PCR+ chimpanzees and 0% gorillas (33.3% EV-A76, EV-D111, EV-B110)				PCR
Harvala et al., 2014 [12]	Enteroviruses (types A–D)	13.1% PCR-positive (23.1% EV-A89, 38.5% EV-A119, 38.5% EV-D120)				PCR
Sadeuh-Mba et al., 2014a [13]	Enteroviruses (types A–D)	5.4% PCR-positive (3.5% wild and 15.2% zoo) (EV-A76, EV-123, EV122, CV-A13, EV-A119, E-29, E-15, CV-A24, EV-B82, EV-A71, SA5)				RT-PCR
Van Nguyen et al., 2014 [14]	Enteroviruses (types A–H and J)	98% PCR-positive mandrills, 100% PCR-positive mangabeys, and 50% PCR-positive monkeys (SA5, EV-B110, J (SV6, EV103, EV108)				RT-PCR
Zheng et al., 2010 [15]	HTLV	7.1% HRLV-1/2 reactive		83% reported NHP contact (99% butchers)		Vironostika HTLV-1/2 EIA + Western blot
Calattini et al., 2011 [16]	HTLV and Simian foamy virus	14.3% HTLV-1-positive (60% subtype B, 40% subtype D), 25.7% HTLV-2-positive (subtype B), and 14.3% SFV-positive			Index case: 60-year-old man with no evident signs	Western blot + INNO-LIA and PCR
Guagliardo et al., 2020 [17]	Monkeypox virus (MPXV)	Total: 34.4% IgG; not smallpox-vaxxed (*n* = 63): 6.3% IgG-positive and 1.7% IgM-positive; vaccinated (*n* = 61): 63.9% IgG-positive		Contact with porcupines (65.6%), Gambian rats (56.8%), sun squirrels (28%), and rope squirrels (26.4%)		Orthopoxvirus IgM/IgG ELISA
Steffen et al., 2020 [18]	Marburg virus	11.25% MARV Neut-positive (66.67% MARV ELISA-positive)				MARV neutralization assay (MARV Neut) + confirmatory MARV-glycoprotein (GP) ELISA
WHO, 2018 [19]	Monkeypox virus (MPXV)	Njikwa: 6 suspected and 1 confirmed; Akwaya: 6 suspected; Biyem-Assi: 1 suspected; Bertoua: 1 suspected; Fotokol: 1 suspected	0 dead		Index case: 20-year-old male from Njikwa HD on 14 May 2016, symptoms: fever, generalized vesiculo-pustular rash, and enlarged lymph nodes with no previous history of travel or contact with an animal suspected of having monkeypox	RT-PCR
Pernet et al., 2014 [20]	Nipah virus	Bats: 47.7% anti-NiV-X-Nabs; exposed individuals: 3.1% (57.1% Centre, 14.3% East, 14.3% South West, 14.3% North West); unexposed: 0%		100% of infected persons butchered bats		Vesicular stomatitis virus (VSV)-based pseudoparticle seroneutralization assay
Courgnaud et al., 2004 [21]	Primate T-cell lymphotropic virus (HTLV/STLV) type 1–3	11.2% HTLV-1 and HTLV-2-positive (75% STLV-positive and 4.1% STLV-1 and STLV-3-positive); 15.3% HTLV-2-positive (100% STLV-3-positive)				ELISA + MUREX (line immunoassay, INNO-LIA) HTLV-I+II confirmatory test and tax PCR
Sadeuh-Mba et al., 2014b [22]	Rabies virus (RABV)	74.2% rabid dogs (65.5% in 2010, 71.4% in 2011, 90.9% in 2012, 70.8% in 2013); 0% monkey and pig				Fluorescent antibody test (FAT)
Sadeuh-Mba et al., 2017 [23]	Rabies virus (RABV)	66.0% rabid dogs (68 Centre, 3 Littoral, 8 North West, 14 West, 3 South West, 3 South, and 1 East) and 0% rabid others				Fluorescent antibody test (FAT)
Betsem et al., 2011 [24]	Simian foamy virus (SFV)	Exposed: 26.7% seropositive, 20.7% PCR-positive (Pygmies: 44.7% seropositive and 29% PCR-positive and Bantu: 16.5% seropositive and 16.5% PCR-positive only males) General population: 2% seropositive and 0.2% PCR-positive (Pygmies: 3.9% seropositive and 0.3% PCR-positive and Bantu: 1.2% seropositive and 0.1% PCR-positive)		52% exposed to monkeys and 48% to apes among contact group	Index case (Bad447): male, second degree wounds from a gorilla at 40 years, sampled at 56 years (2009 and 2010), proviral load = 9 per 10^5^ cells	Western blot + nested PCR
Calattini et al., 2004 [25]	Simian foamy virus (SFV)	11.8% PCR-positive				Western blot + nested PCR
Calattini et al., 2007 [26]	Simian foamy virus (SFV)	9.7% seropositive among exposed (24.1% seropositive exposed to apes and 3.6% exposed to monkeys); of these, 90% PCR integrase-positive		24.1% positive cases exposed to apes and 3.6% to monkeys		Western blot + integrase and LTR PCR confirmation
Wolfe et al., 2004 [27]	Simian foamy virus (SFV)	16.3% EIA-positive and 1% by Western blot				EIA + Western blot
Bacterial zoonoses						
Awah Ndukum et al., 2010 [28]	Bovine tuberculosis (*Mycobacterium bovis*)	31% by Ziehl–Neelsen, 51% by Lowenstein–Jensen, 60% by lateral flow assay (45.56% North West and 14.44% West)				Lowenstein–Jensen culture + Ziehl–Neelsen microscopy + lateral flow-based rapid test
Egbe et al., 2017 [29]	Bovine tuberculosis (*Mycobacterium bovis*)	North West: 2.8% [1.9–3.9]; Adamawa: 7.7% [6.1–9.6]; North: 21.3% [15.2–28.4]; Far North: 13.1% [7.7–20.4]				Culturing/ microscopy and deletion analysis, Hain Genotype MTBC, spoligotyping and MIRU-VNTR
Awah-Ndukum et al., 2018a [30]	Brucellosis	Total = 10.8% [8.6–12.4] RBPT-positive and 8.8% [7.1–10.5] iELISA-positive Adamawa = 12.2% [9.4–15.0] RBPT-positive and 11.3% [8.6–14.0] iELISA-positive North = 8.2% [5.8–10.6] RBPT-positive and 6.1% [4.0–8.2] iELISA-positive		Non-specific (46% “Contact with wildlife”)		Rose Bengal plate test (RBPT) + indirect ELISA
Awah-Ndukum et al., 2018b [31]	Brucellosis	Cattle: 3.4% [1.94–4.86] RBPT-positive and 5.93% [4.03–7.83] iELISA-positive Abattoir personnel: 5.6% [1.24–9.96] RBPT-positive and 12.15% [1.25–9.95] ELISA-positive (all male) Pregnant women: 0.28% by both tests				Rose Bengal plate test (RBPT) + IgG iELISA
Musallam et al., 2019 [32]	Brucellosis	North West: 12.6% [7.6–21.9]; Adamawa: 2.3% [1.0–7.0]				iELISA
Kamga et al., 2020 [33]	Brucellosis	Centre: 8.6% [7.7–16.3]; West: 7.2% [6.9–14.3]; South West: 0.5% [0.13–4.01]; South: 4.65% Overall: cattle 9.12% [8.9–14.3], sheep 8.04% [7.5–16.4], goats 1.1% [0.37–2.65], dogs 6.06% [3.1–19.2], pigs 1.87% [0.36–2.59]				Rose Bengal plate test (RBPT) + iELISA
Kelly et al., 2021 [34]	Brucellosis, leptospirosis, Q fever	North West: brucellosis 4.2% [2.5–7.0] pastoral and 5.0% [0.0–10.6] dairy cattle; leptospirosis 30.7% [26.3–35.5] pastoral and 1.7% [0.0–4.9] dairy cattle; Q fever 14.6% [11.8–18.0] pastoral and 0% dairy cattle Adamawa/Vina Division: brucellosis 1.1% [0.5–2.4] pastoral cattle; leptospirosis 35.9% [31.3–40.7] pastoral cattle; Q fever 12.4% [9.6–15.9] pastoral cattle				ID Screen Brucellosis and Q fever Serum Indirect Multi-species ELISA; PrioCHECK L.hardjo Indirect ELISA
Scolamacchia et al., 2010 [35]	Brucellosis, leptospirosis, Q fever	Brucellosis: 3.1% [1.8–4.4]; leptospirosis: 30.4% [27.6–33.2]; Q fever: 31.3% [27.3–35.0]				Brucella cELISA, Leptospira hardjo ELISA, and CHECKIT Q Fever ELISA kit
Ndip et al., 2005 [36]	*Ehrlichia chaffeensis* and *Ehrlichia canis* (human monocytotropic ehrlichiosis (HME))	16% PCR-positive; 33% *Ehrlichia canis* IgG (all had antibody to *E. canis* and *E. chaffeensis* by immunoblot)				IFA + Western immunoblot and RT-PCR
Ndip et al., 2009 [37]	*Ehrlichia chaffeensis* (human monocytotropic ehrlichiosis (HME))	10% PCR-positive		75% of cases had dogs/livestock and 88.9% had ticks		Real-time PCR + IFAT
Abanda et al., 2019 [38]	Ehrlichiosis, rickettsiosis, Lyme disease	*Anaplasma*/*Ehrlichia* spp: 76.1% (88.5% Adamawa, 95% Far North, 77.4% North); *Rickettsia* spp: 14.3% (15.3% Adamawa, 17.8% Far North, 16.2% North); *Borrelia* spp: 17.9% (23.6% Adamawa, 30% Far North, 11.4% North)				PCR
Ndip et al., 2004a [39]	Spotted fever (rickettsiosis: *Rickettsia africae* and *R. conorii*)	6% seropositive				RT-PCR
Ndip et al., 2004b [40]	Spotted fever (rickettsiosis: *Rickettsia africae* and *R. conorii*), chikungunya (CHIKV), yellow fever virus (YFV), dengue (DENV), West Nile virus (WNV), Spondweni virus	32.1% *R. africae* IgM (35.9% among females and 26.1% among males); antibodies CHIKV: 44.0%; YFV: 39.7%; WNV: 26.5%; Spondweni: 27.8%; DENV: 35.9% (DENV-1: 34.2%, -2: 32.1%, -3: 23.9%, -4: 23.5%)				Immunofluorescence and Western immunoblot assays (rickettsiosis) and haemagglutination inhibition (others)
Parasitic zoonoses						
Kouam et al., 2015 [41]	Myiasis (*Cordylobia anthropophaga*)	25.8% [15.5–38.5] positive farms				Skin larvae identification + microscopy
Kouam et al., 2017 [42]	Myiasis (*Cordylobia anthropophaga*)	2.8% [1.50–5.10] animals and 8.9% positive farms				Skin larvae identification + microscopy
Nkouawa et al., 2009 [43]	Paragonimiasis	14.8% ELISA-positive (68% immunoblot-positive), 0% faecal-positive samples				Formalin–ether concentration stool examination method + ELISA and immunoblots + QIAamp DNA Mini Kit
Moyou-Somo et al., 2003 [44]	Paragonimiasis	Paragonimiasis: 2.56% (17% among males and 8% among females)		100% of cases ate crabs		Microscopy
Nkouawa et al., 2010 [45]	Toxocariasis and paragonimiasis	Toxocariasis: 36.3% (50.8% males); paragonimiasis: 14.9% (40% males)				ELISA
Abongwa et al., 2019 [46]	Toxoplasmosis	22.3% IgG, 1.8% IgM, 5.2% IgG and IgM; 1^st^ trimester (29.2% seropositive); 2^nd^ trimester (25.3% seropositive); 3^rd^ trimester (22.9% seropositive)				OnSite Toxo IgG/IgM rapid test (RDT)
Achonduh-Atijegbe et al., 2016 [47]	Toxoplasmosis	38.3% IgG and 3.2% IgM		12.4% cat-owners and 14.9% dog-owners		OnSite Toxo IgG/IgM rapid test (RDT)
Assob et al., 2011 [48]	Toxoplasmosis	42.1% IgG, 6% IgM, 10.8% IgG and IgM				ELISA
Ayeah et al., 2022 [49]	Toxoplasmosis	Women: 72.7% IgG, 1.3% IgM, 80% IgG or IgM, 6% IgG and IgM; Neonates: 55.2% IgG, 8.9% IgM, 23.9% IgG and IgM		Non-specific (58.7% bushmeat consumption and 88% grilled meat; 33.2% cat owners)		Toxo EIA Rapid Labs kit
Kouitcheu et al., 2018 [50]	Toxoplasmosis	35.8% IgG				Indirect solid-phase EIA
Ndamukong-Nyanga et al., 2021 [51]	Toxoplasmosis	22.8% seropositive; 1^st^ trimester (20%); 2^nd^ trimester (32.8%); 3^rd^ trimester (18.0%)				Gold colloidal chromatographic cassette (TOX IgG/IgM RDT)
Nguefack et al., 2016 [52]	Toxoplasmosis	78.6% IgG, 0.9% IgM and IgG		88.2% seropositive among cat-owners vs 78.1%		ELISA
Nguemaïm et al., 2020 [53]	Toxoplasmosis	33.9% IgG, 0.8% IgG and IgM; 1^st^ trimester (37.5%); 2^nd^ trimester (32.1%)		53.1% seropositive among cat owners (*P*-value = 0.013)		Cassette and buffer immune-chromatographic test (RDT) + ELISA (IgG+/IgM−)
Njunda et al., 2011 [54]	Toxoplasmosis	62.7% IgG [53.7–71.7], 0% IgM, 2.7% IgG and IgM; 1^st^ trimester (75.0%); 2^nd^ trimester (60.6%); 3^rd^ trimester (50.0%)				Toxo-IgG ImmunoComb and Toxo-IgM ‘capture’ ELISA
Guemgne Todjom et al., 2019 [55]	Toxoplasmosis	Total: 29.5% IgG, 12% IgM; HIV-positive patients: 47.2% IgG, 22.2% IgM, 11.1% IgG and IgM; HIV-negative patients: 25.6% IgG, 9.7% IgM, 2.4% IgG and IgM		64.62% seropositive among those exposed to cats (*P*-value <0.001)		iELISA
Wam et al., 2016 [56]	Toxoplasmosis	88.7% IgG, 30.9% IgM, 19.6% IgG and IgM				iELISA
Other vector-borne viral zoonoses						
Fokam et al., 2010 [57]	Chikungunya (CHIKV), yellow fever virus (YFV), o'nyong'nyong (ONNV) viruses, dengue virus (DENV-1 to 4), Wesselsbron virus (WSLV), Zika virus, West Nile virus (WNV), Rift Valley fever virus (RVFV), Semliki Forest virus (SFV), Sindbis virus (SINDV); Middleburg virus (MIDV), Tahyna orthobunyavirus (TAHV)	Zika: 37.97%; YFV: 43.03%; DENV-1: 38.0%; DENV-2: 39.2%; DENV-3: 35.3%; DENV-4: 35.4%; WNV: 34.17% WSLV: 41.8%; CHIKV: 34.2%; ONNV: 34.2%; SFV: 26.7%; SINDV: 13.9%; RVFV: 0%; MIDV: 26.6%; TAHV: 0%				Haemagglutination inhibition (HI), complement fixation (CF) tests + confirmatory plaque-reduction neutralization tests (PRNT)
Demanou et al., 2010 [58]	Chikungunya virus (CHIKV), o'nyong'nyong virus (ONNV), dengue virus (DENV)	CHIKV: 51.4% IgM, 49.5% IgM and IgG, 90.5% IgG; ONNV: 0% IgM, 90.5% IgG; DENV: 0% IgM, 0% IgG				IgM capture enzyme immunoassay (MAC-ELISA) and ELISA IgG suspected case: dengue-like symptoms (febrile) a year before the study
Kuniholm et al., 2006 [59]	Chikungunya virus (CHIKV), West Nile virus (WNV), o'nyong'nyong virus (ONNV), yellow fever virus (YFV), dengue-2 (DENV-2), Sindbis virus (SINDV), Tahyna orthobunyavirus (TAHV)	DENV-2: 12.5%, WNV: 6.6%, YFV: 26.9%, CHIKV: 46.5%, ONNV: 47.7%, SINDV: 7.8%, TAHV: 36.3%				Plaque-reduction neutralization tests (PRNT)
González Gordon et al., 2022 [60]	Crimean–Congo haemorrhagic fever virus (CCHFV)	North West: 57.9% [54.4–61.5] pastoral cattle and 6.7% [2.6–16.1] dairy cattle; Adamawa/ Vina Division: 48.9% [45.3–52.6] pastoral cattle				Anti-CCHFV IgG ELISA
Demanou et al., 2014 [61]	Dengue virus (DENV 1, 2, 3, and 4)	Littoral: 0.3% IgM, 61.4% IgG (17% DENV-1, 61.0% DENV-2); North: 0.1% IgM, 24.2% IgG (11.7% DENV-1, 72.3% DENV-2); Centre: 0% IgM, 9.8% IgG (13.6% DENV-1, 66.7% DENV-2)				In-house IgM capture enzyme immunoassay (MAC-ELISA) and ELISA IgG anti dengue virus
Raulino et al., 2022 [62]	Dengue virus (DENV 1–4), Zika virus (ZIKV), West Nile virus (WNV), Usutu virus (USUV), chikungunya virus (CHIKV), and o'nyong'nyong virus (ONNV)	CHIKV_E2: 1.0% [0.66–1.8], ONNV_E2: 0.94% [0.55–1.61], DENV1_NS1: 1.4% [0.94–2.24], DENV2_NS1: 1.6% [1.05–2.41], DENV3_NS1: 1.4% [0.94–2.24], DENV4_NS1: 2.6% [1.89–3.61], USUV_NS1: 4.0% [3.14–5.25], WNV_NS1: 0.8% [0.44–1.43], WNV_DIII: 0.72% [0.39–1.34], ZIKV_NS1: 0.58% [0.29–1.15]				Luminex assay with recombinant proteins CHIKV_E2, ONNV_E2, DENV_NS, USUV_NS1, WNV_NS1, WNV_DIII, and ZIKV_NS1
Galani et al., 2021 [63]	Dengue virus (DENV)	6.89% seropositive (8.33% IgG, 66.67% IgM, 25% NS1 antigen and IgM)				MSL RDT for dengue NS1 antigen and IgG/IgM antibody
Tchetgna et al., 2021 [64]	Dengue virus (DENV)	12.8% DENV-positive: DENV-3 (68.3%), DENV-2 (19.5%), DENV-1 (4.9%)				RT-PCR
Tchuandom et al., 2018 [65]	Dengue virus (DENV)	Total: 14.4% anti-DENV IgM; Far North (12.7%); Adamawa (15.3%); Centre (16.2%), Littoral (20.7%), West (10.3%)				Tell mefast Combo Dengue NS1-IgG/IgM Rapid Test + confirmatory in-house iELISA
Tchuandom et al., 2019 [66]	Dengue virus (DENV)	6.14% DENV-positive (7.7% among females and 4.7% among males) (0.31% DENV-NS1 antigen-positive; 5.20% DENV-NS1 + DENV IgM; 0.62% DENV-NS1 + IgG)				Tell mefast Combo Dengue NS1-IgG/IgM Rapid Test + confirmatory in-house iELISA
Tchuandom et al., 2020 [67]	Dengue virus (DENV)	4.5% DENV-NS1, 12.3% IgM, 6.1% IgG; Total: 24.8% seropositive (24.6% male and 26.9% female)				IgG/IgM/NS1 combo rapid test + in-house iELISA
Yousseu et al., 2018 [68]	Dengue virus (DENV), chikungunya virus (CHIKV), Zika viruses (ZIKV)	DENV: 7.0% (62.5% DENV-1); ZIKV: 0%; CHIKV: 0%				Trioplex real-time RT-PCR
Mouiche et al., 2020 [69]	Dengue virus (DENV-1)	0.3% DENV-1-positive				PCR
Nemg Simo et al., 2018 [70]	Dengue virus (DENV-1)	14.28% DENV-positive (23.1% IgM-positive and 76.9% IgG-positive/DENV-1)			May 2017, a case of dengue serotype 1 was detected and confirmed through routine surveillance in a traveller returning from Kribi, South	MAC-ELISA + Trioplex real-time RT-PCR
Ebogo-Belobo et al., 2022 [71]	Rift Valley fever virus (RVFV)	Sheep: 6.4%; goat: 4.9% (IgG); 0% IgM among both				cELISA
Rissmann et al., 2017 [72]	Rift Valley fever virus (RVFV)	Cattle: 13.5% [11.4–15.7] = Centre (9.09%), Adamawa (11.7%), South (10.87%), Far North (19.12%), North (11.76%); goats/sheep: 3.4% [2.3–4.7] = Centre (4.93%), South (3.43%), Far North (7.14%), North (2.38%), North West (2.86%), West (1.82%), South West (3.33%)				Indirect IgG ΔGn ELISA for small ruminants; ID Vet Competition ELISA + ID Vet IgM Capture ELISA for cattle + confirmatory serum neutralizing test (SNT) and quantitative real-time RT-PCR
Sadeuh-Mba et al., 2018 [73]	Rift Valley fever virus (RVFV), Crimean–Congo haemorrhagic fever virus (CCHFV)	RVFV: 12.4% (6.7% in Lomie, 0% in Abong-Mbang, 9.1% in Messok and 15.9% in Mindourou); CCHFV: 4.4% (3.3% in Lomie, 25.0% in Abong-Mbang, 0% in Messok and 3.4% in Mindourou)				ELISA
Poueme et al., 2019 [74]	Rift Valley fever virus (RVFV)	Sheep: 4.6% [2.7–7.6] IgG (Benoue = 5.6% [3.2–9.4], Faro = 0%, Mayo Rey = 2.3% [0.1–13.5]) and 0% IgM; goats: 2.3% [1.1–4.6] IgG (Benoue = 4.5% [2.1–9], Faro = 0%, Mayo Rey = 0%) and 0% IgM				cELISA
Nemg Simo et al., 2022 [75]	Yellow fever	Total: 360 confirmed [0–14 years = 127; 15–26 years = 226; ≥65 years = 7; male = 226; female = 133] [2010 = 15; 2011 = 30; 2012 = 29; 2013 = 36; 2014 = 33; 2015 = 78; 2016 = 92; 2017 = 19; 2018 = 8; 2019 = 14; 2020 = 6] [Adamawa = 36; Centre = 33; East = 15; Far North = 35; Littoral = 61; North = 47; North West = 45; South = 21; South West = 36; West = 28]				[2010–2019 samples by IgM capture ELISA and 2020 samples by RT-qPCR] + PRNT (plaque reduction neutralization test); suspected case: WHO 2004 “an illness characterized by an acute onset of fever followed by jaundice within 2 weeks of the onset of the first symptom”
Gake et al., 2017 [76]	Zika virus (ZIKV)	Far North (2.0%), North (4.8%), Adamawa (2.0%), East (7.6%), Centre (3.3%), Littoral (10.0%)				EuroImmun Anti-NS1 IgG ELISA + confirmatory seroneutralization

CS, cross-sectional; HD, health district; HTLV, human T-lymphotropic virus; NHP, non-human primate; STLV, simian T-cell lymphotropic virus; TB, tuberculosis; WHO, World Health Organization.

EIA, enzyme immunoassay; HD, health district; HPAIV, highly pathogenic avian influenza virus; HTLV, human T-lymphotropic virus; NHP, non-human primate; STLV, simian T-cell lymphotropic virus; WHO, World Health Organization.

**Table:** 

References
[1] Njouom R, Aubin J-T, Bella AL, Demsa BM, Rouquet P, Gake B, et al. Highly pathogenic avian influenza virus subtype H5N1 in ducks in the Northern part of Cameroon. Vet Microbiol 2008;130:380–4. https://doi.org/10.1016/j.vetmic.2008.02.006.
[2] Monamele CG, Y P, Karlsson EA, Vernet M-A, Wade A, Okomo M-CA, et al. Evidence of exposure and human seroconversion during an outbreak of avian influenza A(H5N1) among poultry in Cameroon. Emerg Microbes Infect 2019;8:186–96. https://doi.org/10.1080/22221751.2018.1564631.
[3] Wade A, Taïga T, Fouda MA, MaiMoussa A, Jean Marc FK, Njouom R, et al. Highly pathogenic avian influenza A/H5N1 Clade 2.3.2.1c virus in poultry in Cameroon, 2016-2017. Avian Pathol 2018;47:559–75. https://doi.org/10.1080/03079457.2018.1492087.
[4] Wade A, Dickmu Jumbo S, Zecchin B, Fusaro A, Taiga T, Bianco A, et al. Highly Pathogenic Avian Influenza A(H5N8) Virus, Cameroon, 2017. Emerging Infectious Diseases 2018;24:1367–70. https://doi.org/10.3201/eid2407.172120.
[5] Snoeck C, OJ A, A S, MP O, AG O, AA O, et al. Serological evidence of pandemic (H1N1) 2009 virus in pigs, West and Central Africa. Veterinary Microbiology 2015;176:165–71.
[6] Larison B, Njabo KY, Chasar A, Fuller T, Harrigan RJ, Smith TB. Spillover of pH1N1 to swine in Cameroon: an investigation of risk factors. BMC Vet Res 2014;10:55. https://doi.org/10.1186/1746-6148-10-55.
[7] Njabo KY, Fuller TL, Chasar A, Pollinger JP, Cattoli G, Terregino C, et al. Pandemic A/H1N1/2009 influenza virus in Swine, Cameroon, 2010. Vet Microbiol 2012;156:189–92. https://doi.org/10.1016/j.vetmic.2011.09.003.
[8] Njouom R, Yekwa EL, Cappy P, Vabret A, Boisier P, Rousset D. Viral etiology of influenza-like illnesses in Cameroon, January-December 2009. J Infect Dis 2012;206:S29–35.
[9] De Nys HM, Kingebeni PM, Keita AK, Butel C, Thaurignac G, Villabona-Arenas C-J, et al. Survey of Ebola Viruses in Frugivorous and Insectivorous Bats in Guinea, Cameroon, and the Democratic Republic of the Congo, 2015-2017. Emerging Infectious Diseases 2018;24:2228–40. https://doi.org/10.3201/eid2412.180740.
[10] Steffen I, Lu K, Yamamoto LK, Hoff NA, Mulembakani P, Wemakoy EO, et al. Serologic Prevalence of Ebola Virus in Equatorial Africa. Emerg Infect Dis 2019;25:911–8. https://doi.org/10.3201/eid2505.180115.
[11] Harvala H, Sharp CP, Ngole EM, Delaporte E, Peeters M, Simmonds P. Detection and genetic characterization of enteroviruses circulating among wild populations of chimpanzees in Cameroon: relationship with human and simian enteroviruses. J Virol 2011;85:4480–6. https://doi.org/10.1128/JVI.02285-10.
[12] Harvala H, Van Nguyen D, McIntyre C, Ahuka-Mundeke S, Ngole EM, Delaporte E, et al. Co-circulation of enteroviruses between apes and humans. J Gen Virol 2014;95:403–7. https://doi.org/10.1099/vir.0.059048-0.
[13] Sadeuh-Mba SA, Bessaud M, Joffret M-L, Endegue Zanga M-C, Balanant J, Mpoudi Ngole E, et al. Characterization of Enteroviruses from non-human primates in cameroon revealed virus types widespread in humans along with candidate new types and species. PLoS Negl Trop Dis 2014;8:e3052. https://doi.org/10.1371/journal.pntd.0003052.
[14] Van Nguyen D, Harvala H, Ngole EM, Delaporte E, Woolhouse MEJ, Peeters M, et al. High rates of infection with novel enterovirus variants in wild populations of mandrills and other old world monkey species. J Virol 2014;88:5967–76. https://doi.org/10.1128/JVI.00088-14.
[15] Zheng H, Wolfe ND, Sintasath DM, Tamoufe U, LeBreton M, Djoko CF, et al. Emergence of a novel and highly divergent HTLV-3 in a primate hunter in Cameroon. Virology 2010;401:137–45. https://doi.org/10.1016/j.virol.2010.03.010.
[16] Calattini S, Betsem E, Bassot S, Chevalier SA, Tortevoye P, Njouom R, et al. Multiple retroviral infection by HTLV type 1, 2, 3 and simian foamy virus in a family of Pygmies from Cameroon. Virology 2011;410:48–55. https://doi.org/10.1016/j.virol.2010.10.025.
[17] Guagliardo SAJ, Monroe B, Moundjoa C, Athanase A, Okpu G, Burgado J, et al. Asymptomatic Orthopoxvirus Circulation in Humans in the Wake of a Monkeypox Outbreak among Chimpanzees in Cameroon. Am J Trop Med Hyg 2020;102:206–12. https://doi.org/10.4269/ajtmh.19-0467.
[18] Steffen I, Lu K, Hoff NA, Mulembakani P, Okitolonda Wemakoy E, Muyembe-Tamfum J-J, et al. Seroreactivity against Marburg or related filoviruses in West and Central Africa. Emerg Microbes Infect 2020;9:124–8. https://doi.org/10.1080/22221751.2019.1709563.
[19] World Health Organization. Monkeypox – Cameroon n.d. https://www.who.int/emergencies/disease-outbreak-news/item/05-june-2018-monkeypox-cameroon-en [accessed 1 December 2022].
[20] Pernet O, Schneider BS, Beaty SM, Lebreton M, Yun TE, Park A, et al. Evidence for henipavirus spillover into human populations in Africa. Nat Commun 2014;5.
[21] Courgnaud V, Van Dooren S, Liegeois F, Pourrut X, Abela B, Loul S, et al. Simian T-Cell Leukemia Virus (STLV) Infection in Wild Primate Populations in Cameroon: Evidence for Dual STLV Type 1 and Type 3 Infection in Agile Mangabeys (Cercocebus agilis). Journal of Virology 2004;78:4700–9. https://doi.org/10.1128/JVI.78.9.4700-4709.2004.
[22] Sadeuh-Mba SA, Besong L, Demanou M, Loul S, Nchare A, Njouom R. Laboratory data of dog rabies in southern Cameroon from 2010 to 2013. BMC Res Notes 2014;7:905. https://doi.org/10.1186/1756-0500-7-905.
[23] Sadeuh-Mba SA, Momo JB, Besong L, Loul S, Njouom R. Molecular characterization and phylogenetic relatedness of dog-derived Rabies Viruses circulating in Cameroon between 2010 and 2016. PLoS Negl Trop Dis 2017;11:e0006041. https://doi.org/10.1371/journal.pntd.0006041.
[24] Betsem E, Rua R, Tortevoye P, Froment A, Gessain A. Frequent and Recent Human Acquisition of Simian Foamy Viruses Through Apes’ Bites in Central Africa. PLoS Pathog 2011;7:e1002306. https://doi.org/10.1371/journal.ppat.1002306.
[25] Calattini S, Nerrienet E, Mauclère P, Georges-Courbot M-C, Saïb A, Gessain A. Natural simian foamy virus infection in wild-caught gorillas, mandrills and drills from Cameroon and Gabon. J Gen Virol 2004;85:3313–7. https://doi.org/10.1099/vir.0.80241-0.
[26] Calattini S, Betsem EBA, Froment A, Mauclère P, Tortevoye P, Schmitt C, et al. Simian foamy virus transmission from apes to humans, rural Cameroon. Emerg Infect Dis 2007;13:1314–20. https://doi.org/10.3201/eid1309.061162.
[27] Wolfe ND, Switzer WM, Carr JK, Bhullar VB, Shanmugam V, Tamoufe U, et al. Naturally acquired simian retrovirus infections in central African hunters. Lancet 2004;363:932–7. https://doi.org/10.1016/S0140-6736(04)15787-5.
[28] Awah Ndukum J, Kudi AC, Bradley G, Ane-Anyangwe IN, Fon-Tebug S, Tchoumboue J. Prevalence of bovine tuberculosis in abattoirs of the littoral and Western highland regions of cameroon: a cause for public health concern. Vet Med Int 2010;2010:495015. https://doi.org/10.4061/2010/495015.
[29] Egbe NF, Muwonge A, Ndip L, Kelly RF, Sander M, Tanya V, et al. Molecular epidemiology of Mycobacterium bovis in Cameroon. Sci Rep 2017;7:4652. https://doi.org/10.1038/s41598-017-04230-6.
[30] Awah-Ndukum PA J, Mouiche, MMM, Bayang, HN, Ngwa, VN, Assana, E, Feussom, KJM, Manchang, TK, Zoli. Seroprevalence and Associated Risk Factors of Brucellosis among Indigenous Cattle in the Adamawa and North Regions of Cameroon 2018;2018.
[31] Awah-Ndukum J, Mouiche MMM, Kouonmo-Ngnoyum L, Bayang HN, Manchang TK, Poueme RSN, et al. Seroprevalence and risk factors of brucellosis among slaughtered indigenous cattle, abattoir personnel and pregnant women in Ngaoundéré, Cameroon. BMC Infect Dis 2018;18:611. https://doi.org/10.1186/s12879-018-3522-x.
[32] Musallam I, Ndour AP, Yempabou D, Ngong C-AC, Dzousse MF, Mouiche-Mouliom M-M, et al. Brucellosis in dairy herds: A public health concern in the milk supply chains of West and Central Africa. Acta Trop 2019;197:105042. https://doi.org/10.1016/j.actatropica.2019.105042.
[33] Kamga RMN, Silatsa BA, Farikou O, Kuiate J-R, Simo G. Detection of Brucella antibodies in domestic animals of southern Cameroon: Implications for the control of brucellosis. Vet Med Sci 2020;6:410–20. https://doi.org/10.1002/vms3.264.
[34] Kelly RF, Jennings A, Hunt J, Hamman SM, Mazeri S, Nkongho EF, et al. The epidemiology of bacterial zoonoses in pastoral and dairy cattle in Cameroon, Central Africa. Zoonoses Public Health 2021;68:781–93. https://doi.org/10.1111/zph.12865.
[35] Scolamacchia F, Handel IG, Fèvre EM, Morgan KL, Tanya VN, Bronsvoort BM de C. Serological patterns of brucellosis, leptospirosis and Q fever in Bos indicus cattle in Cameroon. PLoS One 2010;5:e8623. https://doi.org/10.1371/journal.pone.0008623.
[36] Ndip LM, Ndip RN, Esemu SN, Dickmu VL, Fokam EB, Walker DH, et al. Ehrlichial infection in Cameroonian canines by Ehrlichia canis and Ehrlichia ewingii. Vet Microbiol 2005;111:59–66. https://doi.org/10.1016/j.vetmic.2005.08.010.
[37] Ndip LM, Labruna M, Ndip RN, Walker DH, McBride JW. Molecular and clinical evidence of Ehrlichia chaffeensis infection in Cameroonian patients with undifferentiated febrile illness. Ann Trop Med Parasitol 2009;103:719–25. https://doi.org/10.1179/000349809 × 12554106963753.
[38] Abanda B, Paguem A, Abdoulmoumini M, Kingsley MT, Renz A, Eisenbarth A. Molecular identification and prevalence of tick-borne pathogens in zebu and taurine cattle in North Cameroon. Parasit Vectors 2019;12:448. https://doi.org/10.1186/s13071-019-3699-x.
[39] Ndip LM, Fokam EB, Bouyer DH, Ndip RN, Titanji VPK, Walker DH, et al. Detection of Rickettsia africae in patients and ticks along the coastal region of Cameroon. Am J Trop Med Hyg 2004;71:363–6.
[40] Ndip LM, Bouyer DH, Travassos Da Rosa APA, Titanji VPK, Tesh RB, Walker DH. Acute spotted fever rickettsiosis among febrile patients, Cameroon. Emerg Infect Dis 2004;10:432–7. https://doi.org/10.3201/eid1003.020713.
[41] Kouam MK, Meutchieye F, Nguafack TT, Miegoué E, Tchoumboué J, Theodoropoulos G. Parasitic fauna of domestic cavies in the western highlands of Cameroon (Central Africa). BMC Vet Res 2015;11:288. https://doi.org/10.1186/s12917-015-0605-4.
[42] Kouam MK, Meutchieye F, Miegoue E, Nguafack TT, Tchoumboue J, Teguia A. Prevalence and husbandry-related risk factors of myiasis in domestic cavies in the western highlands of Cameroon. Epidemiol Infect 2017;145:339–46. https://doi.org/10.1017/S0950268816002466.
[43] Nkouawa A, Okamoto M, Mabou AK, Edinga E, Yamasaki H, Sako Y, et al. Paragonimiasis in Cameroon: molecular identification, serodiagnosis and clinical manifestations. Transactions of the Royal Society of Tropical Medicine and Hygiene 2009;103:255–61. https://doi.org/10.1016/j.trstmh.2008.09.014.
[44] Moyou-Somo R, Kefie-Arrey C, Dreyfuss G, Dumas M. An epidemiological study of pleuropulmonary paragonimiasis among pupils in the peri-urban zone of Kumba town, Meme Division, Cameroon. BMC Public Health 2003;3:40. https://doi.org/10.1186/1471-2458-3-40.
[45] Nkouawa A, Sako Y, Itoh S, Kouojip-Mabou A, Nganou CN, Saijo Y, et al. Serological studies of neurologic helminthic infections in rural areas of southwest cameroon: toxocariasis, cysticercosis and paragonimiasis. PLoS Negl Trop Dis 2010;4:e732. https://doi.org/10.1371/journal.pntd.0000732.
[46] Abongwa LE, Signang A, Tibi S, Ngenwi A. Socio-demographic and Obstetric Variations of T. gondii and HIV-1 Co-infection among Pregnant Women in Cameroon. Journal of Advances in Microbiology 2019:1–11. https://doi.org/10.9734/jamb/2019/v16i330122.
[47] Achonduh-Atijegbe OA, Mfuh KO, Mbange AHE, Chedjou JP, Taylor DW, Nerurkar VR, et al. Prevalence of malaria, typhoid, toxoplasmosis and rubella among febrile children in Cameroon. BMC Infect Dis 2016;16:658. https://doi.org/10.1186/s12879-016-1996-y.
[48] Assob JCN, Njunda AL, Nsagha DS, Kamga HL, Weledji PE, Che VB. Toxoplasma antibodies amongst HIV/AIDS patients attending the University Teaching Hospital Yaounde in Cameroon. African Journal of Clinical and Experimental Microbiology 2011;12. https://doi.org/10.4314/ajcem.v12i3.6.
[49] Ayeah JN, Oladokun A, Sumbele IUN, Ilesanmi AO, Bekindaka ON. Seroprevalence of Gestational and Neonatal Toxoplasmosis as well as Risk Factors in Yaoundé, Cameroon. J Parasitol Res 2022;2022:6406259. https://doi.org/10.1155/2022/6406259.
[50] Kouitcheu LBM, Tchakounte C, Bonsi ST, Etoa F-X. Prevalence of Toxoplasma gondii and Associated Risk Factors among Pregnant Women Attending Hospital Centers in Penka-Michel, Cameroon. Journal of Scientific Research and Reports 2018:1–11. https://doi.org/10.9734/JSRR/2018/33061.
[51] Ndamukong-Nyanga JL, Linda KF, Lucile STC, Dolly-Misper DT. The Severity of Malaria and Toxoplasmosis Co-Infections among Pregnant Women in Yaounde, Cameroon. Undefined 2021.
[52] Nguefack C, Meumeu I, Ngaba G, Kongnyuy E, Njamen T, Halle ekane G, et al. Prevalence and Factors Associated with Toxoplasma Gondii Immunization among Pregnant Women in Douala – Cameroon. Women s Health Issues 2016;5:1000248.
[53] Nguemaïm NF, Takang WA, Dobgima WP, Guebidiang BM, Foumane P, Kamga FHL. Seroprevalence of Toxoplasma gondii infection and associated risk factors among pregnant women attending antenatal clinic at the Bamenda Regional Hospital, Cameroon. Séroprévalence de l'infection à Toxoplasma Gondii et Des Facteurs de Risque Associés Chez Les Femmes Enceintes En Visites Prénatales à l'Hôpital Régional de Bamenda Au Cameroun 2020;21:123–31.
[54] Njunda AL, Nsagha DS, Assob J-CN, Kamga H-FF, Tafili RT, Achidi EA. Seroepidemiology of toxoplasmosis in pregnant women attending the University Teaching Hospital in Yaounde, Cameroon. Int J Health Res 2011;4:1–9.
[55] Guemgne Todjom F, Makou Tsapi E, Gamago GA, Vignoles P, Wabo Pone J, Djuikwo Teukeng FF. Seroprevalence of Toxoplasmosis and associated risk factors in pregnant women at the Protestant Hospital, Mbouo-Bandjoun, Cameroon. Afr J Clin Exp Microbiol 2019;20:221–30.
[56] Wam EC, Sama LF, Ali IM, Ebile WA, Aghangu LA, Tume CB. Seroprevalence of Toxoplasma gondii IgG and IgM antibodies and associated risk factors in women of child-bearing age in Njinikom, NW Cameroon. BMC Res Notes 2016;9:406.
[57] Fokam EB, Levai LD, Guzman H, Amelia PA, Titanji VPK, Tesh RB, et al. Silent circulation of arboviruses in Cameroon. East Afr Med J 2010;87:262–8. https://doi.org/10.4314/eamj.v87i6.63085.
[58] Demanou M, Antonio-Nkondjio C, Ngapana E, Rousset D, Paupy C, Manuguerra J-C, et al. Chikungunya outbreak in a rural area of Western Cameroon in 2006: A retrospective serological and entomological survey. BMC Res Notes 2010;3:128. https://doi.org/10.1186/1756-0500-3-128.
[59] Kuniholm MH, Wolfe ND, Huang CY-H, Mpoudi-Ngole E, Tamoufe U, LeBreton M, et al. Seroprevalence and distribution of Flaviviridae, Togaviridae, and Bunyaviridae arboviral infections in rural Cameroonian adults. Am J Trop Med Hyg 2006;74:1078–83.
[60] González Gordon L, Bessell PR, Nkongho EF, Ngwa VN, Tanya VN, Sander M, et al. Seroepidemiology of Crimean-Congo Haemorrhagic Fever among cattle in Cameroon: Implications from a One Health perspective. PLoS Negl Trop Dis 2022;16:e0010217. https://doi.org/10.1371/journal.pntd.0010217.
[61] Demanou M, Pouillot R, Grandadam M, Boisier P, Kamgang B, Hervé JP, et al. Evidence of dengue virus transmission and factors associated with the presence of anti-dengue virus antibodies in humans in three major towns in Cameroon. PLoS Negl Trop Dis 2014;8:e2950. https://doi.org/10.1371/journal.pntd.0002950.
[62] Raulino R, Thaurignac G, Keita AK, Esteban A, Goumou S, Diallo R, et al. Seroprevalence of IgG Antibodies Against Multiple Arboviruses in Bats from Cameroon, Guinea, and the Democratic Republic of Congo. Vector Borne Zoonotic Dis 2022;22:252–62. https://doi.org/10.1089/vbz.2021.0076.
[63] Galani BRT, Mapouokam DW, Simo FBN, Mohamadou H, Chuisseu PDD, Njintang NY, et al. Investigation of dengue-malaria coinfection among febrile patients consulting at Ngaoundere Regional Hospital, Cameroon. J Med Virol 2021;93:3350–61. https://doi.org/10.1002/jmv.26732.
[64] Tchetgna HDS, Yousseu FS, Kamgang B, Tedjou A, Mccall PJ, Wondji CS. Concurrent Circulation of Dengue Serotype 1, 2 and 3 among Acute Febrile Patients in Cameroon. Int J Infect Dis 2022;116:S125.
[65] Tchuandom SB, Tchouangueu TF, Antonio-Nkondjio C, Lissom A, Djang JON, Atabonkeng EP, et al. Seroprevalence of dengue virus among children presenting with febrile illness in some public health facilities in Cameroon. Pan Afr Med J 2018;31:177. https://doi.org/10.11604/pamj.2018.31.177.16390.
[66] Tchuandom SB, Tchadji JC, Tchouangueu TF, Biloa MZ, Atabonkeng EP, Fumba MIM, et al. A cross-sectional study of acute dengue infection in paediatric clinics in Cameroon. BMC Public Health 2019;19:958. https://doi.org/10.1186/s12889-019-7252-9.
[67] Tchuandom SB, Lissom A, Ateba GHM, Tchouangueu TF, Tchakounte C, Ayuk AR, et al. Dengue virus serological markers among potential blood donors: an evidence of asymptomatic dengue virus transmission in Cameroon. Pan Afr Med J 2020;36:185. https://doi.org/10.11604/pamj.2020.36.185.22128.
[68] Yousseu FBS, Nemg FBS, Ngouanet SA, Mekanda FMO, Demanou M. Detection and serotyping of dengue viruses in febrile patients consulting at the New-Bell District Hospital in Douala, Cameroon. PLoS One 2018;13:e0204143. https://doi.org/10.1371/journal.pone.0204143.
[69] Mouiche MMM, Ntumvi NF, Maptue VT, Tamoufe U, Albert B, Ngum Ndze V, et al. Evidence of Low-Level Dengue Virus Circulation in the South Region of Cameroon in 2018. Vector Borne Zoonotic Dis 2020;20:314–7. https://doi.org/10.1089/vbz.2019.2531.
[70] Nemg Simo FB, Sado Yousseu FB, Evouna Mbarga A, Bigna JJ, Melong A, Ntoude A, et al. Investigation of an Outbreak of Dengue Virus Serotype 1 in a Rural Area of Kribi, South Cameroon: A Cross-Sectional Study. Intervirology 2018;61:265–71. https://doi.org/10.1159/000499465.
[71] Ebogo-Belobo JT, Sadeuh-Mba SA, Mveng-Sanding GMA, Chavely GM, Groschup MH, Mbacham WF, et al. Serological evidence of the circulation of the Rift Valley fever virus in sheep and goats slaughtered in Yaoundé, Cameroon. Vet Med Sci 2022.
[72] Rissmann, M., Eiden, M., Wade, A., Poueme, R., Abdoulkadiri, S., Unger, et al. Evidence for enzootic circulation of Rift Valley fever virus among livestock in Cameroon. Acta Tropica 2017;172:7–13.
[73] Sadeuh-Mba SA, Yonga Wansi GM, Demanou M, Gessain A, Njouom R. Serological evidence of rift valley fever Phlebovirus and Crimean-Congo hemorrhagic fever orthonairovirus infections among pygmies in the east region of Cameroon. Virol J 2018;15:63. https://doi.org/10.1186/s12985-018-0977-8.
[74] Poueme R, Stoek F, Nloga N, Awah-Ndukum J, Rissmann M, Schulz A, et al. Seroprevalence and associated risk factors of rift valley fever in domestic small ruminants in the North Region of Cameroon. Vet Med Int 2019;2019.
[75] Nemg FBS, Abanda NN, Yonga MG, Ouapi D, Samme IE, Djoumetio MD, et al. Sustained circulation of yellow fever virus in Cameroon: an analysis of laboratory surveillance data, 2010-2020. BMC Infect Dis 2022;22:418. https://doi.org/10.1186/s12879-022-07407-1.
[76] Gake B, Vernet MA, Leparc-Goffart I, Drexler JF, Gould EA, Gallian P, et al. Low seroprevalence of Zika virus in Cameroonian blood donors. Braz J Infect Dis 2017;21:481–3. https://doi.org/10.1016/j.bjid.2017.03.018.

### 3.2 Viral zoonotic diseases reported

Several of the studies that reported viral zoonotic diseases investigated these in animals and only a few involved human participants (8/27). Studies of zoonotic infections found a broad range of diseases, with a sample size ranging from 35 (in a family-based investigation of human T-lymphotropic virus (HTLV) subtype 3 and other simian retroviruses in a Bakola Pygmy 60-year-old man who had a positive test: 14.3% tested positive for HTLV-1 and 25.7% HTLV-2, and 14.3% for simian foamy virus (SFV)) to 4478 (domestic birds sampled between 2016 and 2018 in Adamawa, Centre, South, and West Regions for highly pathogenic avian influenza A (H5N1) with a positivity rate of 7.3%). Between 2009 and 2012, some studies reported low proportions of swine flu (H1N1) among commercial pigs and birds (0.6–5.6%) and humans (5.2%) in the Centre, Littoral, North, and West Regions. In a study by Steffen et al., 1.3% of the 240 healthy individuals tested in the South Region, tested positive for antibodies against Ebola virus (formerly Ebola Zaire). A majority of dogs (66%) presenting at veterinary clinics in the Centre, East, Littoral, North West, and West Regions (2010–2016) tested positive for rabies virus. A study conducted in October 2017 in the Centre and South Regions among 125 healthy individuals working at a primate sanctuary and living in villages around the sanctuary, reported evidence of monkeypox infection; 34.4% of individuals who had never received the smallpox vaccine compared to 6.3% of those who had previously received the smallpox vaccine, tested positive for antibodies (IgG) against monkeypox.

### 3.3 Bacterial zoonotic diseases reported

Overall, seven bacterial zoonoses were reported in the included studies (*n* = 13). The prevalence of brucellosis among cattle and other livestock has predominantly been investigated, and a majority in the Adamawa region (5/6), with a period prevalence of 1.3% in Vina Division, Adamawa (January–November 2013) to 12.6% in Bamenda, North West (February 2017–January 2018) among pastoral cattle. In two studies conducted by Ndip and colleagues in 2001 and 2003, patients presenting at the Cameroon Development Corporation Central Tiko Clinic and Mount Mary Health Centre in the South West tested positive for antibodies against spotted fever (rickettsiosis) attributed to *Rickettsia africae* and *Rickettsia conorii*, with a prevalence of 6.0% (7/118) and 32.1% (75/234), respectively. Compared to a cross-sectional survey in April–May 2008 of a sample of cattle from abattoirs in the North West and West (35 292), with a reported bovine tuberculosis positivity rate of 45.6% and 14.4%, respectively, a similar study performed in April 2012–October 2013 in a reduced sample (2346) resulted in the following proportions: North West (2.8%), Adamawa (7.7%), North (21.3%), Far North (13.1%).

### 3.4 Parasitic zoonotic diseases reported

For studies that reported toxoplasmosis prevalence, positivity rates for prior infections (IgG) were greater than 20%. Most of these studies focused on pregnant women visiting different clinics in their first, second, or third trimester. In one such study performed in April–June 2014, of 643 pregnant women (age 27.1 ± 2.51 years, 39.5% second trimester and 50.7% third trimester) presenting at Penka-Michel and Menoua clinics, West Region, 35.8% were seroreactive. Similarly, a January–April 2015 study at Douala Regional Hospital and two private clinics, revealed that 78.6% were seroreactive. In June 2019–May 2020, Ayeah and colleagues investigated for the presence of antibodies against *Toxoplasma gondii* in neonates in two hospitals in the Centre Region via cord blood specimens, and 55.2% tested positive for IgG, 8.9% IgM, and 23.9% IgG and IgM. In addition, five of the 16 included studies reported other parasitic zoonotic diseases: 14.8% of individuals who consumed crabs in Tombel Health District and 2.6% of pupils at a primary school in Kumba, South West Region had detectable levels of *Paragonimus* spp.

### 3.5 Other vector-borne viral zoonotic diseases reported

**Figure 2:**
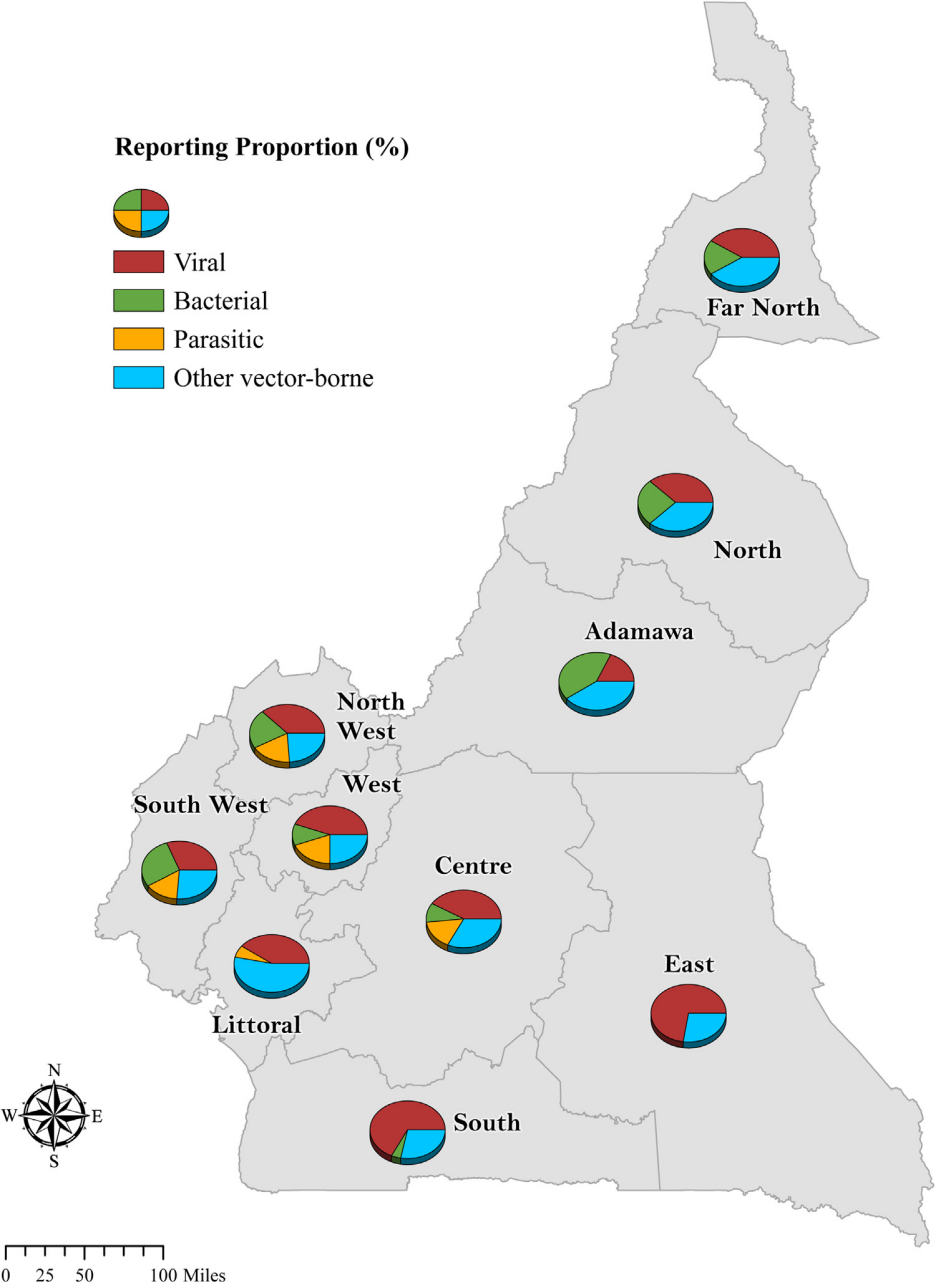
Reporting proportions of different zoonosis groups by region in Cameroon (2000–2022).

The majority of studies that reported vector-borne zoonoses focused on dengue virus fever (14/21) in humans (mostly febrile patients) and one in frugivorous and insectivorous bats across all 10 regions. The prevalence of dengue fever varied for studies reporting serum IgG and IgM: Littoral (0.3% IgM, 61.4% IgG), North (0.1% IgM, 24.2% IgG), Centre (0% IgM, 9.8% IgG) in September 2006–December 2007; and Adamawa (4.6% IgM, 0.6% IgG) among febrile patients in October 2019–January 2020. The second most reported vector-borne zoonosis was chikungunya virus fever (6/21). The prevalence was relatively higher for chikungunya, at more than 34.0% across all studies. A 2013–2014 investigation by Rissmann and co-authors across nine regions showed evidence of an enzootic prevalence of Rift Valley fever of 13.5% (95% CI: 11.4–15.7%) in cattle (Centre (9.1%), Adamawa (11.7%), South (10.9%), Far North (19.1%), North (11.8%)) and of 3.4% (95% CI: 2.3–4.7%) in sheep/goats (Centre (4.9%), South (3.4%), Far North (7.1%), North (2.4%), North West (2.9%), West (1.8%), South West (3.3%)). Evidence of Zika virus was reported across three studies, with a prevalence of 40.0% among febrile patients in the South West (Fako Division Provincial Hospital Annex, Mount Mary Health Centre, and Cameroon Development Corporation Central Clinic), in August–October 2015 among blood donors in the Far North (2.0%), North (4.8%), Adamawa (2.0%), East (7.6%), Centre (3.3%), and Littoral (10.0%), and more recently in bats sampled in the Centre (Obala and Yaoundé), South (Campo), and East (Mambele) (0.6%) regions. Similarly, West Nile virus was reported among febrile patients presenting at Mount Mary Health Center and Cameroon Development Corporation Central Clinic, South West at separate periods, with a prevalence of 26.5% and 34.2% in 2001 and 2010, respectively (Figure 2).

### 3.6 Meta-analysis by zoonotic disease to assess heterogeneity

Four of the reported diseases – brucellosis, dengue, influenza, and toxoplasmosis – had the number of studies sufficient for a power of ≥53%. It was expected that the reported prevalence across studies would vary for reasons other than sampling error (diagnostic tool, study population, and study period), which explains the SD = 6 and use of a random-effects model. The forest plots (Supplementary Material File S3, Figures S1–S4) show the pooled prevalence, *I*^2^ statistics, and *P*-values associated with these. The pooled prevalence estimates should not be interpreted as representative of the burden of the specified zoonotic diseases across the national territory because of the expected variation in meta-analyses of prevalence studies. For the meta-analysis, all four pooled prevalence estimates among those surveyed in the different studies (febrile and non-ill community members) were associated with an *I*^2^ statistic greater than 75% (high inter-study heterogeneity) and *P* < 0.01: brucellosis (random-effects pooled estimate proportion, ES 0.05%, 95% CI 0.03–0.07; *I*^2^ = 90.91%; *n* = 6), dengue (ES 0.13%, 95% CI 0.06–0.22; *I*^2^ = 98.90%; *n* = 12), avian and swine influenza virus (ES 0.10%, 95% CI 0.04–0.20; *I*^2^ = 98.29%; *n* = 8), and toxoplasmosis (ES 0.49%, 95% CI 0.35–0.63; *I*^2^ = 98.39%; *n* = 11).

### 3.7 Risk of bias analysis

**Figure 3:**
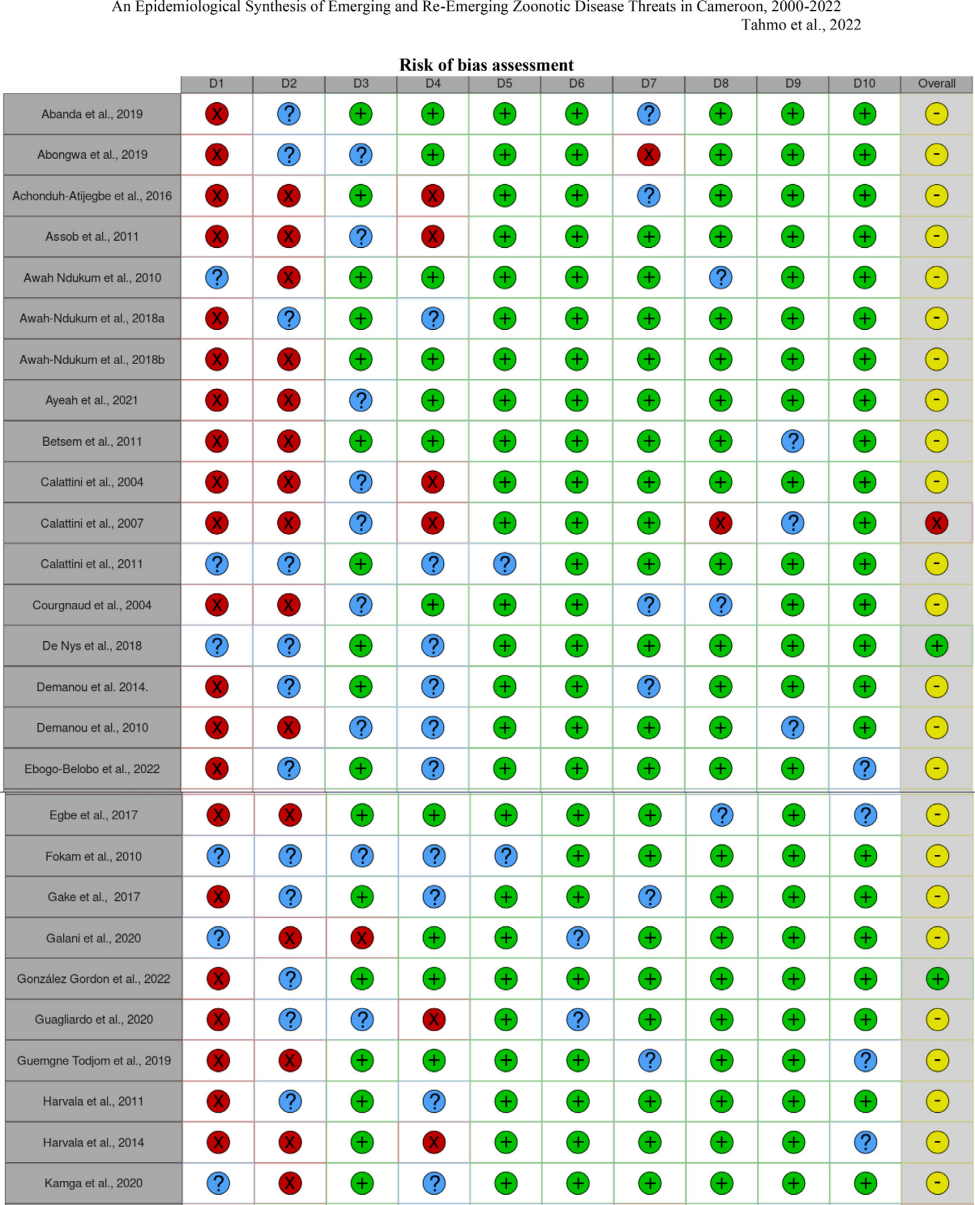

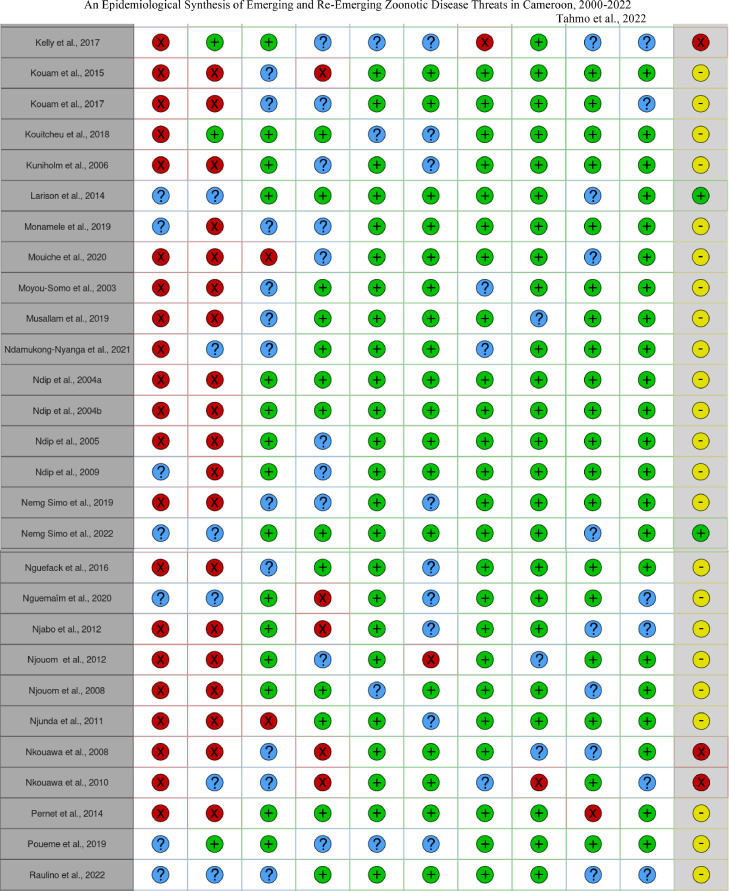

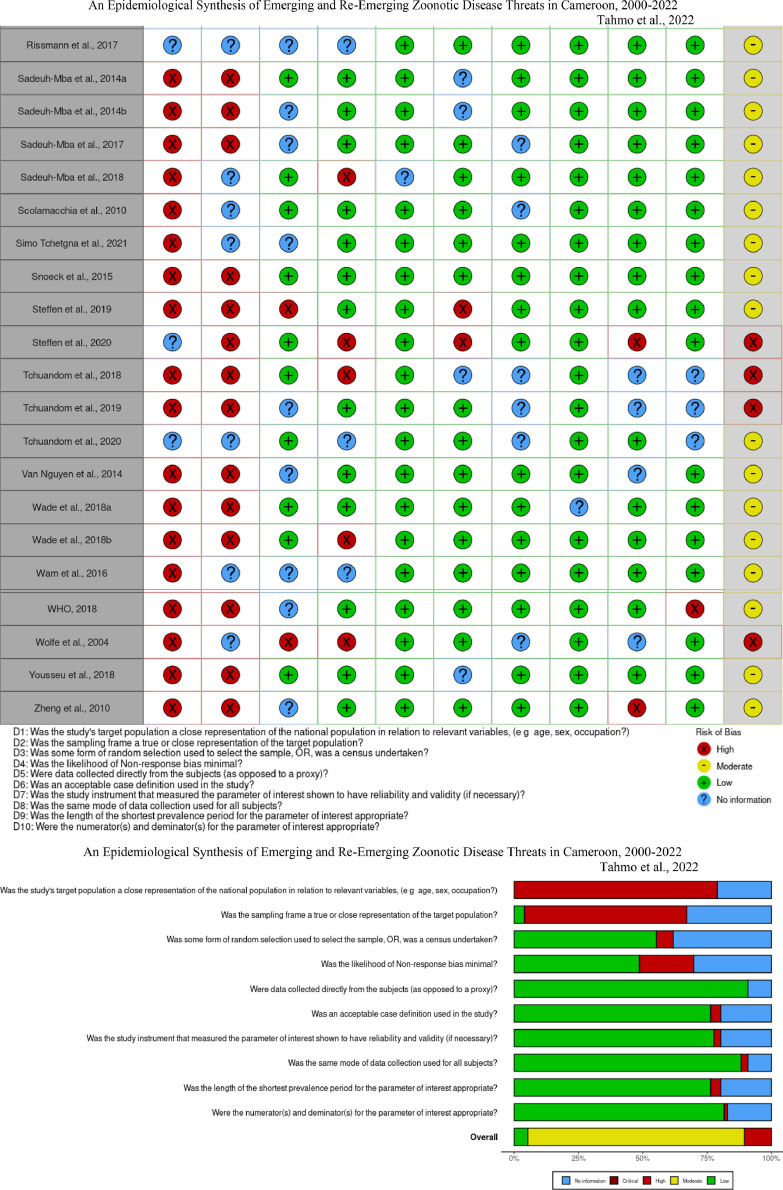
Risk of bias assessment.

Figure 3 summarizes the results of the risk of bias analysis for the included studies. Of the 76 studies, eight had a high risk of bias, 64 a moderate risk, and four a low risk. For studies with a high risk of bias, they failed almost entirely in terms of external generalizability, and all of the included studies where information was provided were not representative of the national population in terms of demographics like age, sex, or occupation. All of the studies used the proper numerator and denominator in estimating prevalence, and all except two studies used appropriate diagnostic tools that have been shown to have acceptable validity and reliability. The studies checked ‘Yes’ for a majority of the questions regarding internal validity; 373 out of 456 (81.8%).

## 4 Discussion

Although epidemiological data on various zoonotic diseases from the different health districts are found scattered across the literature, to date there are no clearly available specific details of the burden of zoonotic diseases in Cameroon. This systematic review provides a one-stop resource for understanding, through an evidence-based systematized approach, the threat of pathogen exposures of Cameroonian communities related to zoonotic diseases. Overall, 35 unique zoonotic diseases reported in at least one region of Cameroon between January 2000 and May 2022 were identified. This concerted effort is consistent with the National Program for the Prevention and Fight Against Emerging and Re-emerging Zoonoses (NPPFERZD) and One Health Cameroon (national zoonoses programme) goal to have a unified and informed approach to risk management (community awareness, capacity strengthening, and prioritization) of zoonotic threats [7].

From the studies that were reviewed, there is clearly a pattern in the number of research groups that have investigated particular groups of zoonotic diseases. Emphasis has been placed on an array of viral zoonoses: over the second decade of the study period of interest (2011–2022), more studies focused on vector-borne zoonoses; in the first decade, more studies focused on other viral zoonoses. Besides these, studies have been limited to brucellosis and toxoplasmosis, and other zoonotic threats have been investigated quite sparingly. The south-eastern portion of Cameroon and scattered parts of the Centre and North West Regions covered by the Congo Basin rainforest (16 674 023 hectares) teem with a unique biodiversity, including about 335 mammal species, 874 bird species, and 218 amphibian species [21], [22]. Additionally, Cameroon is characterized by live market networks where livestock and other food products are exchanged. This clustering and movement within and between regions is a catalyst that drives the spread of diseases through interactions of infected and susceptible people at the community level, including the spatial overlap of non-human primate density with human activities, which transfers to trucking routes where bushmeat is sold [23]. A majority of the studies included in this review were hospital-based cross-sectional studies, and among those investigating animals, most studied cattle and swine in select farms or markets in the North, Adamawa, and North West regions. Also, epidemiological data that are available through the District Health Information System of the Ministry of Public Health are mostly case counts as reported by the respective health districts [24]. These are passive surveillance results; the actual burden is likely underestimated, as suggested by the current study findings. Increased vigilance for zoonotic disease events is indicated in order to better inform risk management efforts.

This systematic review also identified a need for more robust population research and case finding, consistent with other systematic reviews conducted by different research groups for specific zoonotic diseases like leptospirosis and monkeypox, or groups of zoonotic diseases in general [13], [14], [15],25]. Differentiating zoonotic diseases as emerging or re-emerging is time and location specific. Emerging zoonoses would include those that have either never occurred before or have affected a small proportion of individuals and whose incidence is increasing, whereas re-emerging zoonoses include those that are well known but whose incidence has significantly increased or the host–environment–vector interaction has changed. It would be misleading to interpret the epidemiological data from this systematic review as indicative of the trend of the different zoonotic diseases that have been indexed.

For the pathogens with at least five studies, heterogeneity was greater than 75%. This is expected with a meta-analysis of prevalence studies because of the difference in the sampling frame, sampling technique, sampling size, diagnostic tool, and differing biomarkers reported as a result [26].

### 4.1 Limitations

Although an evidence-based systematic approach was used to synthesize the literature and abstract the epidemiological data related to zoonoses in Cameroon, the findings should be interpreted with caution. First, the search strategy applied included the names of Cameroon and WHO-designated priority zoonotic diseases in addition to general terms. As such, it is possible that some literature databases did not return articles where the words zoonotic disease or animal disease or vector-borne or cross-species transmission or interspecies transmission were not used. Nonetheless, care was taken to use indexed/controlled terms for each database, such as MeSH terms in PubMed. Second, based on the risk of bias analysis, 73 out of the 76 studies included utilized a sampling frame that was not representative of the target population and none were representative of the national demographics. Third, some zoonotic diseases of known aetiology such as hepatitis E virus infection, fascioliasis, and microsporidiosis were excluded; human African trypanosomiasis was excluded because of indistinctness with the subspecies (zoonotic *Trypanosoma brucei rhodesiense* versus non-zoonotic *Trypanosoma brucei gambiense*) in some studies [27], [28], [29]. Severe acute respiratory syndrome coronavirus 2 (SARS‑CoV‑2) was intentionally not indexed, as while animal–human–animal cycles have occurred, the pandemic is dominated by human-to-human transmission, confounding observation of this dynamic [30]. Fourth, although additional evidence was obtained from the grey literature, including country reports (District Health Information Software 2 and the National Zoonoses Program), the data were not included in this review because of ethical concerns and unbalanced data by reporting year. Fifth, social disturbance, including conflict, drought, population migration, and other stresses on communities, are incompletely accounted for in the literature and are factors important to the health security risk. Last, all of the studies included were cross-sectional studies and, as such, these are only point and period prevalence data. There were no data on the severity or duration of the infections, and no inference can be made about the demographic risk factors for the respective diseases.

### 4.2 Conclusions

This systematic review bridges some existing gaps in the understanding of the landscape of zoonoses and exposes critical gaps in the surveillance and reporting of zoonotic diseases in Cameroon. There is evidence of viral, bacterial, and parasitic zoonoses across the territory, many of which have epidemic potential. The SARS-CoV-2 pandemic and monkeypox epidemic underscore the critical role of pandemic preparedness. Therefore, there is a need to study definitive reservoir–vector–acquisition associations and to strengthen passive surveillance systems and reporting of active or sentinel surveillance findings. In Cameroon, an improved understanding of specific groups and communities at higher risk than others for emerging and re-emerging zoonotic spillover events will allow careful prioritization of limited resources for better One Health risk management.

## Appendix Supplementary materials

**File S1** PRISMA checklist. Image, application 1

## Footnotes

Supplementary material associated with this article can be found, in the online version, at doi:10.1016/j.ijregi.2022.12.001.
