# Supplementary material for: An epidemiological synthesis of emerging and re-emerging zoonotic disease threats in Cameroon, 2000–2022: a systematic review
Source: IJID Reg. 2022 Dec 17;7:84–109. doi: 10.1016/j.ijregi.2022.12.001 (PMC10050484; doi:10.1016/j.ijregi.2022.12.001)

Figure 3: Forest plot for brucellosis

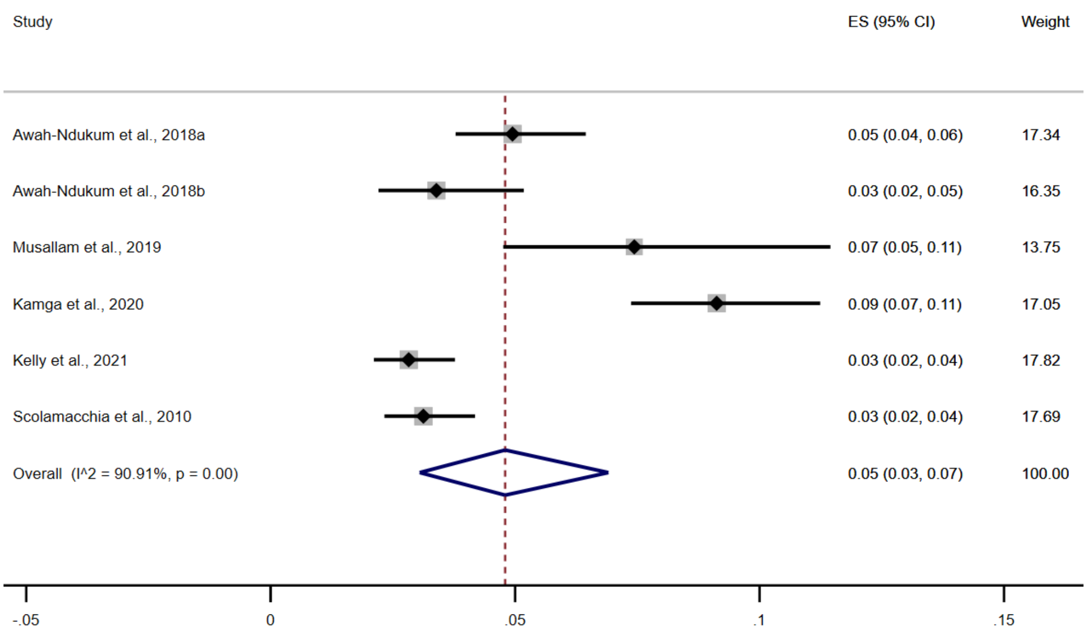

Figure 4: Forest plot for dengue fever

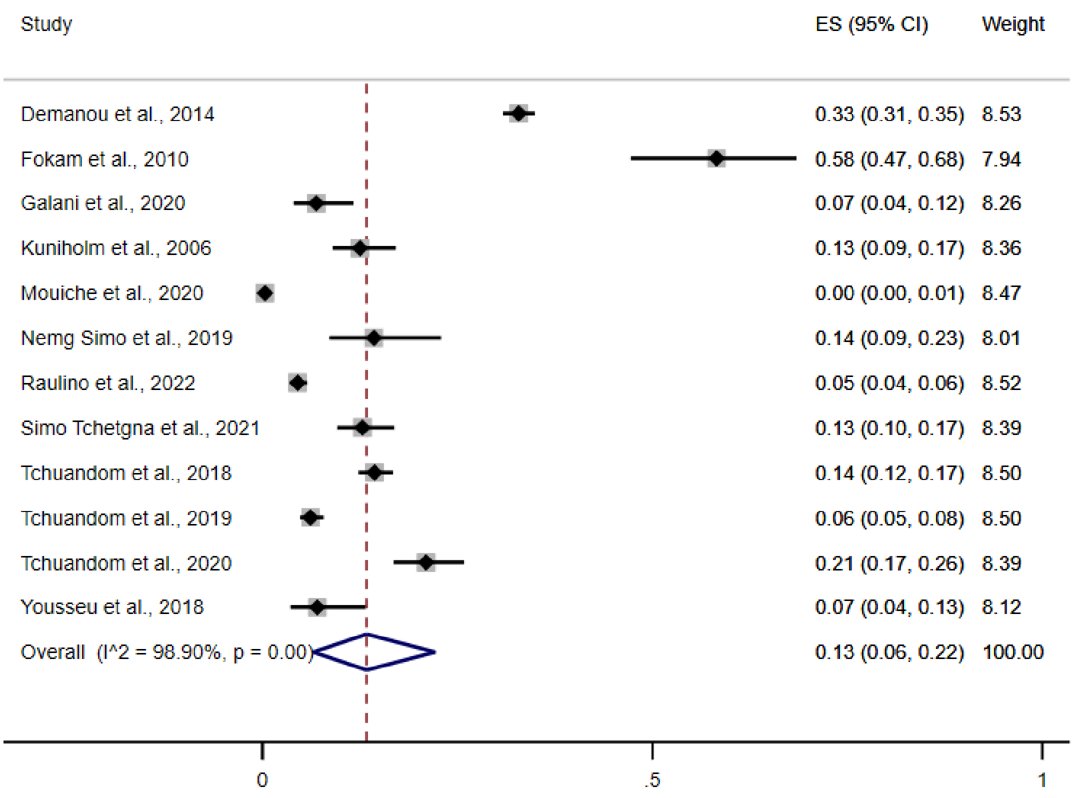

Figure 5: Forest plot for avian & swine influenza virus

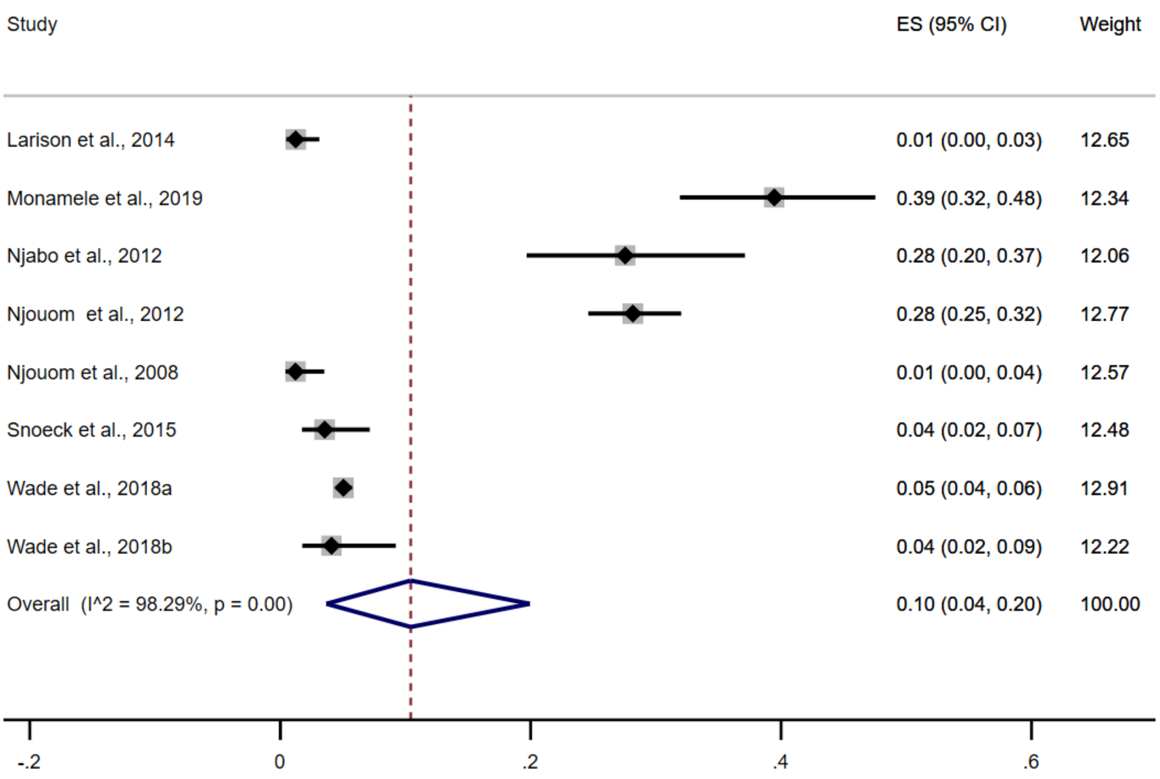

Figure 6: Forest plot for toxoplasmosis

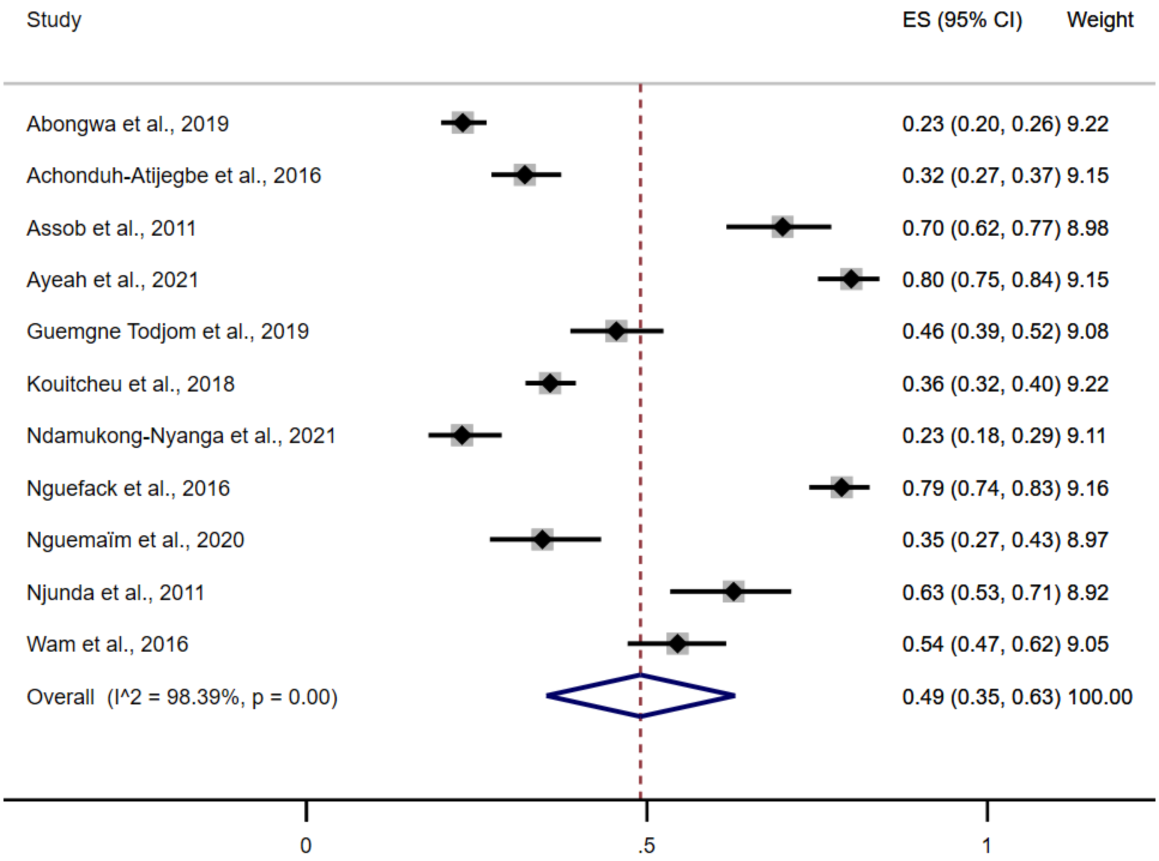

Supplement: Supplementary file 1 — File S1 PRISMA checklist. [file mmc1.pdf]
